# Electrode performance in hybrid electrocoagulation and biochar adsorption systems for olive mill wastewater treatment

**DOI:** 10.1371/journal.pone.0360811

**Published:** 2026-10-26

**Authors:** Ahmad Jamrah, Aseel Al-Khraisha, Khaldoun Shatanawi, Tharaa M. Al-Zghoul, Emad K. Al-Karablieh

**Affiliations:** 1 Department of Civil Engineering, School of Engineering, The University of Jordan, Amman, Jordan; 2 Water, Energy and Environment Center, The University of Jordan, Amman, Jordan; 3 Department of Agricultural Economics and Agribusiness, School of Agriculture, The University of Jordan, Amman, Jordan; National Research and Innovation Agency, INDONESIA

## Abstract

Olive mill wastewater (OMW) is a major environmental concern in olive-producing regions because of its high organic load and phytotoxic phenolic content, which limit its safe discharge or reuse. This study aimed to develop and evaluate an integrated electrocoagulation–adsorption (ECAD) treatment using olive pomace (OP) biochar and to determine how electrode material influences the performance and cost of the combined process. Specifically, the study characterized OP biochar produced by pyrolysis and evaluated its adsorption behavior toward total phenols (TPh) and soluble chemical oxygen demand (COD_soluble_), compared Al- and Fe-based EC and their integrated ECAD performance, and theoretically determined biochar dosages and estimated the operational cost of both configurations. The produced biochar exhibited a BET surface area of 135.23 m^2^/g, a mesoporous structure with an average pore width of 21.95 Å, a negative zeta potential of −29.89 mV, and a carbon content of 67.21%, confirming the formation of a carbon-rich and porous adsorbent. Isotherm analysis showed that TPh adsorption was best described by the Langmuir model, with a maximum adsorption capacity of 84.74 mg/g, whereas COD_soluble_ was best fitted by the Dubinin–Radushkevich (D–R) model. Standalone EC achieved comparable TPh and COD_soluble_ removal using Al and Fe electrodes (about 52–55%). However, the integrated systems showed a strong electrode-dependent response. The Al-ECAD system achieved maximum removals of 70.69% TPh and 71.39% COD_soluble_, while the Fe-ECAD system reached 67.40% and 66.33%, respectively, at the lowest biochar dosage, with removal declining at higher dosages. The estimated operational costs were 4.25 and 5.30 US$/m^3^ for Al- and Fe-based systems, respectively. The novelty of this work lies in demonstrating that the effectiveness of biochar-assisted EC is strongly dependent on electrode–biochar compatibility, identifying electrode selection as a key design factor for ECAD treatment of OMW.

## Introduction

Water scarcity and water pollution are severe global issues associated with population growth, industrialization, and urbanization [1]. Previous reports suggested that 1.7–2.4 billion people had inhabiting in water-scarce conditions, while polluted water contributed for around 1.6 million deaths in 2018 [2–4]. These impacts have had a significant effect on both the quantity and quality of freshwater resources, particularly in water-scarce areas. Among the sources of highly polluted wastewater, agro-industrial production is particularly affected, with significant environmental impact from the olive oil industry [5,6].

The olive oil sector has been heavily concentrated in the Mediterranean region, which represents more than 75% of world production and produces around 30 million m^3^ of olive mill waste water (OMW) annually, an estimated 40 million m^3^ worldwide [7,8]. In Jordan, more than 21,000 tons of oil are produced from olives alongside about 45,000 tons of solid waste, primarily olive pomace (OP, “Jift”), and about 96,000 m^3^ of OMW (“Zibar”) [9]. The process of production includes four stages: cleaning of olives, crushing and malaxation, separation of oil from solid phase, and final separation from the liquid phase [10,11]. OMW is the most challenging fraction of these waste streams due to its high volume, liquid form and high pollutant content, among the originating waste streams. It was reported that nearly 1.3 m^3^ of wastewater was generated per ton of processed olives [12].

The environmental problem related with OMW is due to its complex, acidic and recalcitrant nature. OMW may have chemical oxygen demand (COD) concentrations as high as 320 g/L, biological oxygen demand (BOD) concentrations up to 132 g/L and phenolic content from 0.5 to 24 g/L [8,13,14]. It can also have acidic pH values between 2.2 and 6 as well as high levels of suspended and dissolved solids [8,13]. The biodegradability is decreased by the high content of phenolic compounds and other organic inhibitors, which also complicate biological processing [8,13]. As a result, the release of OMW into the land without any treatment can lead to soil sealing, plant growth inhibition, surface and groundwater contamination, and harm to aquatic and terrestrial ecosystems [15,16]. These effects are more pronounced in Jordan where an arid climate and scarce freshwater resources make the environmental sound management of OMW paramount [17,18]. Therefore, the OMW needs to be properly treated prior to disposal or application.

Different treatment techniques have been studied for OMW treatment such as membrane filtration [19], coagulation–flocculation [20], advanced oxidation processes [21], as well as aerobic and anaerobic biological treatments [22]. Nevertheless, the effectiveness of a single process for the treatment of OMW is usually restricted by OMW's complex nature. Biological processes are limited by OMW low biodegradability and toxic properties, as well as by the long retention times that may be required and by the post-treatment before discharge [22]. Aerobic biological treatments may also have relatively high energy and operational demands, while physicochemical treatments may be energy-intensive and relatively low in COD removal [23,24]. Membrane processes can deliver high-quality effluent, but are vulnerable to membrane fouling and high operation and maintenance demands [23,24]. Such limitations imply that the conventional treatment may not be able to stably produce the desired quality of effluent. As a result, combined treatment approaches have now attracted more attention because the integration of complementary techniques could potentially improve pollutant removal and overcome the inherent limitations of individual treatment steps.

Electrocoagulation (EC) has been consequently considered as a promising physicochemical treatment method because it produces coagulant agents in situ by electrochemical oxidation of electrodes [25,26]. The released metal ions induce the destabilization and agglomeration of suspended and dissolved pollutants, and the hydrogen evolution on the cathode may aid in flotation of the formed flocs. In contrast to traditional chemical coagulation, EC has number of advantages, such as minimal reliance on externally supplied chemical coagulants, uncomplicated operation, fast start-up, relatively small sludge production, and efficient elimination of fine colloidal and organic pollutants [27–29]. Thus, these features render EC an excellent candidate for the treatment of industrial wastewaters that require high amounts of reagents in normal chemical treatment coupled with further sludge handling.

However, EC alone may not always provide adequate elimination of highly soluble and persistent organic compounds found in OMW. This constraint gives a reason to couple EC with other suitable polishing treatments. Earlier research has demonstrated that combined treatment allows for better removals compared to the single processes such as EC–adsorption systems have been reported to accomplish 20% more removals than EC only [11,30]. This is especially the case with OMW, as EC facilitates the destabilization and elimination of suspended and coagulated contaminants, while adsorption can further address rad residual dissolved organic matter and phenolic components.

Adsorption has been recognized as an effective polishing step due to its high efficiency, low energy consumption, and operational simplicity, with reported removal efficiencies reaching up to 99.9% for certain pollutants [31]. Biosorbents made from agricultural wastes have recently been considered as inexpensive substitutes to traditional adsorbents and at the same time they provide the possibility for waste agriculture to be reused [31,32]. Different types of agricultural wastes such as pomegranate seeds, corn straw, rice husk, and by-products of processing of olive fruits have been tested as bio-based/biosorbents for the treatment of wastewaters [33,34]. In this regard, OP constitutes an especially appealing, readily available material in Jordan as it is produced in abundance by the olive oil industry, the same which yields OMW. Hence, the transformation of OP to biochar might offer a locally sourced adsorbent for extraction of residual organic and phenolic pollutants and at the same time provide the means for valorizing an agricultural waste stream [33,34].

The necessity for treating OMW effectively is also highlighted by the regulations on discharge and/or the reuse of the treated water. In Jordan, the permissible COD concentrations according to the JS 202/2007 and the relevant instructions were 150 mg/L and 100 mg/L for use in agricultural irrigation and discharge in sewerage systems, respectively, while the allowable concentration of phenolic compounds was 0.002 mg/L [17]. International recommendations, such as those of the Food and Agriculture Organization (FAO), allow for COD levels not exceeding 125 mg/L when irrigation of sensitive crops is anticipated and phenolic levels below 1 mg/L, depending on the nature of the reuse [35]. These stringent limits suggest that addressing the bulk organics load may not be adequate and that a method for the removal of soluble, and possibly phytotoxic, compounds are also needed. Hence, a treatment process that can achieve good bulk removal followed by polishing to handle dissolved contaminants is desirable.

The EC and adsorption have been studied separately and sequentially for wastewater treatment, OMW included [36], yet significant challenges are to be addressed when implementing both processes with locally sourced OP biochar and various electrode materials. Previous EC studies demonstrated that electrode material and electrochemical conditions substantially influenced treatment performance, electrode consumption, corrosion, passivation, and contaminant removal [37,38]. Recent studies have confirmed the applicability of aluminium based EC to treat wastewater and high removal efficiency for color, turbidity, organic matter and suspended solids has been cited [39]. Still, aluminum electrodes are prone to fouling, and passivation due to accumulation of contaminants and formation of oxide layers at the electrodes surfaces, which reduces the electrodes activity and consequently the efficiency of the process [28]. These findings highlight the importance of electrode selection when applying EC to complex wastewater matrices.

Nonetheless, the relative performances of the Al and Fe electrodes in a combined EC–adsorption system for the treatment of OMW using biochar derived from OP have not been unequivocally determined. Especially, it is not well known that to what extent the choice of electrode affects the subsequent adsorption polishing stage, and therefore the overall treatment performance. Thus, in this work, a combined EC-adsorption process using an OP-based biochar as adsorbent for the treatment of OMW is presented and aluminum and iron electrodes are comparatively assessed in terms of their influence on the removal of pollutants and the total treatment efficiency. The novelty of this study was the integration of locally derived OP biochar with EC while systematically assessing the role of electrode material in the treatment of highly loaded OMW, thereby providing an integrated approach for pollutant removal and agricultural waste valorization.

## Materials and methods

### Chemicals and reagents

All the reagents and chemicals utilized in the experiment were of analytical reagent grade. All reagents, including sodium hydroxide (1 N NaOH), the COD kit HR (0-15,000 mg/L), sulfuric acid (1 N H_2_SO_4_), ethanol (C_2_H_6_O), hydrochloric acid (0.1 M HCl), and sodium chloride (NaCl) were purchased from Alnoorien Est. for Laboratories & Scientific Supplies, Amman, Jordan and the COD kit was also obtained from Hilmi Abu Sham and Partners Company, Amman, Jordan. Folin–Ciocalteu reagent, anhydrous sodium carbonate (Na_2_CO_3_), and dried gallic acid (GA) were obtained from Sidor Company, Irbid, Jordan.

### OMW sampling collection and pretreatment

Samples of OMW were gathered from an olive mill using the three-phase extraction procedure in Mahis, Jordan (31.9904° N, 35.7725° E). Between November and December 2024, sampling took place during the season of olive harvesting. Notably, such mill operations involve no addition of chemicals. The harvested OMW was brought from the storing pit directly in clean 20-L plastic bottle to the laboratory and the samples were kept at 4 °C until they were analyzed. All studies were carried out at the Environmental Engineering Laboratory, Department of Civil Engineering, University of Jordan. The OMW samples were collected with the consent of olive mill operators, with logistical support from the University of Jordan.

As a result of the high total suspended solid (TSS) content of the OMW, a pretreatment process was performed prior to the analysis and the further adsorption studies. The samples were let to settle first, and then the settled solids were taken away through decantation. Then the supernatant was filtered through a cloth filter. Following pretreatment stock OMW had total COD (COD_total_) and total phenol (TPh) amounts of 67,800 mg/L and 944.62 mg/L as GA, correspondingly. A pretreatment similar to this was used in order to isolate a representative liquid phase for the subsequent adsorption studies. Such a decantation and subsequent cloth filtration procedure has also been described for treatment of OMW [23].

### Physicochemical analysis

The following physicochemical parameters were monitored to assess treatment method efficiency: pH, temperature, electrical conductivity, turbidity, TPh, soluble chemical oxygen demand (COD_soluble_), COD_total_, and TSS. The pH and temperature were determined by using a HANNA pH meter (HI 2002-02, Hanna Instruments). Electrical conductivity was determined with a Delta OHM instrument (HD 3406.2) and was expressed in mS/cm. Turbidity was measured with a Velp Scientifica TB1 turbidity Meter and the results are in nephelometric turbidity units (NTU). An analytical balance (BEL Engineering) was employed in the mass measurements. The COD levels were measured using a HACH Lange DR 3900 spectrophotometer (Hach Company, Loveland, USA) and the results were expressed as mg/L. The analysis was performed as described in Jamrah et al. [6] and Baird et al [40]. Just like the COD_soluble_, the COD_total_ was also measured and the required analytical procedure.

The TPh content was assessed using the "Folin-Ciocalteu Method," in mg/L as gallic acid equivalent, setting the wavelength of the spectrophotometer at 765 nm. The analysis was carried out according to the method described [41]. TSS were measured by the filtration of a homogenized sample on a 0.45 μm pore size, pre-weighed glass-fiber filter. The filter was then dried at 103–105 °C for 30 minutes, and the TSS concentration was calculated based on the mass gain of the filter [40]. The removal efficiency (*R*, %) was calculated by the following equation [41]:


$$\begin{array}{c} R\left(\%\right)=\frac{\left(c_{0}-c_{e}\right)}{c_{e}}\times \text{1}\text{0}\text{0} \end{array}$$ (1)

where (*C*_*0*_) and (*C*_*e*_) are the initial and the final concentrations (mg/L).

### Analytical measurements and quality control

Analytical measurements were performed to describe the initial physicochemical characteristics of the OMW and concentrations of target pollutants, COD_soluble_, and TPh concentration. All the experiments were carried out in duplicate in order to evaluate the reproducibility of the measurements. The analytical precision was estimated by the Relative Standard Deviation (*RSD*), which was calculated using the following equation [42]:


$$\begin{array}{c} RSD(\%)=\frac{SD}{X_{avg}}\times \text{1}\text{0}\text{0} \end{array}$$ (2)

Where *SD* is the standard deviation and *X*_avg_ is the average equilibrium concentration. During the considered equilibrium concentration range, the calculated *RSD* values were under 7.2% for TPh and 3.1% for soluble COD. These figures were consistent with the established limits of 12% for TPh and 9.6% for soluble COD as documented in the Standard Methods [40].

### Olive mill solid waste sampling and preparation of biochar

Samples of olive mill solid waste (OMSW) were collected at an olive mill in Ajloun, Jordan, during October – December 2023, and transported to the Chemistry Laboratory of University of Jordan for biochar preparation. The obtained OMSW was washed repeatedly with distilled water to eliminate remaining impurities and dried in an oven (MATEST A005-08 KIT) at 50 °C for 24 h. The clean olive pomace samples were dried for 5 h in the oven at 105 °C, then the material was sieved through 4.75-mm sieve to separate residual olive leaves, stones and other coarse materials. This separation ensured that only the desired material was used for biochar production. For the heat treatment, samples of 100 grams of the sorted OMSW were wrapped in aluminum foil and subjected to heating in a muffle furnace” “Nabertherm, Lilienthal, Germany” at pyrolysis temperature of 600°C for a duration time of 5 hours. The pyrolysis was performed under ambient air conditions, and the samples were wrapped in aluminum foil to minimize oxidation. After pyrolysis, the biochar was pulverized using a Silver Crest grinder (SC-1880, Hong Kong) and then sieved by means of an electric shaker (Electromagnet A059-02 KIT, Arcore, Italy). The 0.075 mm biochar fraction was then collected and kept in glass bottles for further use.

### Biochar characterization

The biochar yield was expressed as the dry mass of biochar produced on a dry OMSW basis. The biochar pH was measured after mixing biochar with distilled water at a solid-to-liquid ratio of 3:50 (w/w) and the suspension was agitated with a magnetic stirrer for 2 h. The pH was determined by using a pH meter (HI 2002-02, Hanna Instruments Ltd., Bedfordshire, UK) [43]. The EC was also determined by EC meter (CON 6, LaMotte Co., Washington, DC, USA). The ash content was determined according to a modified ASTM method [43,44]. About 5 g of biochar (oven-dried) were weighed and then heated at 750 °C for 6 h. The samples were then cooled to room temperature in desiccators and reweighed. The ash content was determined from the residual mass after thermal treatment [6]. The biochar zeta potential was also measured at the pH of OMW relevant to the application under consideration. Fourier-transform infrared spectroscopy (FTIR) and scanning electron microscopy (SEM) analyses were conducted as described previously [6]. X-ray diffraction (XRD) analysis was performed on AERIS X-ray diffractometer (Malvern Panalytical, Almelo, The Netherlands).

### Experimental procedure

The treatment was a two-stage sequential process, where EC served as the first stage and biochar adsorption as the second. Batch adsorption of biochar was also performed for the removal of COD_soluble_ and TPh prior to combining it in the EC process, in order to check the adsorption performance of biochar.

### Electrocoagulation experiment

Prior to each run, the reactor and electrodes were cleaned with 0.1 N HCl to eliminate possible surface contaminants, rinsed with distilled water and air dried. Then using a graduated cylinder, the pretreated OMW was poured into the reactor. The conditions for operating EC experiments were taken from the optimum condition cited in two previous works of Jamrah et al. [5,6]. The electrode distance was constant (1 cm) and six electrodes were inserted for each system (Fe and Al). The electrodes measured (10 cm × 6 cm × 2 mm). The current densities used were 15.1104 mA/cm^2^ for the Fe electrodes and 12.4173 mA/cm^2^ for the Al electrodes. The equivalent reaction times were 53.49 min for the Fe-EC process and 45.6 min for the Al-EC process. The starting pH of the OMW was adjusted to 4 prior to the application of the electric current.

The power supply was run at 4.352 A for Fe and 3.567 A for the Al system, which translates to the current densities for each respective system. The applied geometrical area of the electrode for the calculation was 288 cm^2^ (4 cm^2^ × 6 cm^2^ × 2 mm^2^ × 6). At the end of each EC run, the power supply was switched off, and the samples under treatment were moved in 1,000-mL beakers and sealed with Parafilm to avoid contamination. The samples were then left at 4 °C for 24 h in order to complete settling of the formed flocs and separation of the sludge and treated water. The settling time was sufficient for a clear supernatant [6,40]. The supernatant was decanted and the concentrations of TPh and COD_soluble_ were determined.

### Adsorption isotherm experiment

Two sets of samples were employed to test the adsorptive capacity of biochar produced from OP for the elimination of COD_soluble_ and TPh. The experiments were carried out with real OMW and synthetic solutions obtained from a GA stock solution, used as a proxy of phenolic compounds. In every trial, 25 ml of the solution was placed in a 250 ml flask. To ascertain the initial concentration of COD_soluble_, the OMW samples were filtered prior to the adsorption trials. Then, 0.05 g of the synthesized biochar was weighed with the electronic analytical balance and added to each flask. The initial COD_soluble_ concentrations of diluted OMW and TPh were 700–12000 mg/L and 25–500 mg/L, respectively.

The flasks were covered with Parafilm seal to avoid spillage during shaking and were laid on an orbital shaker at 150 rpm at room temperature for 24 h for the adsorption equilibrium. The agitation rate of 150 rpm was chosen to ensure uniform suspension and optimal dispersion of the biochar particles in the aqueous medium, thereby minimizing mass transfer limitation without potential damage to the adsorbent particles, as reported in the literature [45–47]. The samples were filtered through 0.45 μm pore size syringe filters after the adsorption time. The filtrates were then analyzed for final concentrations of COD_soluble_ and TPh. All experimental conditions, other than the initial concentrations of diluted OMW and TPh solutions, were taken from the recent study.

### Adsorption isotherm models plot

Adsorption isotherms were plotted by employing the experimentally found C_e_ (concentration at equilibrium in the solution) and the related q_e_ (amount adsorbed at equilibrium). Four isotherms were considered: (1) Experimental isotherm, (2) Langmuir model, (3) Freundlich model, and (4) Dubinin–Radushkevich (D-R) isotherm. The experimental equilibrium adsorption capacity (q_experimental)_ was determined from the following Equation [48]:


$$\begin{array}{c} q_{experimental}=\frac{(C_{i}-C_{e})\times V}{M} \end{array}$$ (3)

where *C*_*i*_ and *C*_*e*_ are the adsorbate concentrations at initial and equilibrium conditions (mg/L), *V* is the volume of the treated solution (L), *M* is the mass of adsorbent (g).

The equilibrium adsorption capacities from Langmuir (q_langmuir model_), Freundlich (q_freundlich model_), and D-R (q_D-R model_) isotherm models were calculated by Equations (4–6), respectively [48]:


$$\begin{array}{c} q_{langmuirmodel}=\frac{q_{max}K_{L}C_{e}}{\text{1}+K_{L}C_{e}} \end{array}$$ (4)

$$\begin{array}{c} q_{freundlichmodel}=K_{F}{C_{e}}^{\frac{\text{1}}{n}} \end{array}$$ (5)

$$\begin{array}{c} q_{DRmodel}=q_{m}\text{exp}\left({-K}_{DR}\varepsilon^{\text{2}}\right),where\varepsilon =RTln(\text{1}+\frac{\text{1}}{ce}) \end{array}$$ (6)

The relevant model parameters were obtained from the linearized forms of the corresponding isotherms. The maximum adsorption capacity (q_m_, mg/g) and the Langmuir constant (K_L_, L/mg) related to the adsorption energy were obtained from the linear plot of C_e_/q_e_ versus C_e_ for the Langmuir model. In the case of the Freundlich model, the adsorption capacity constant (K_F_, (mg/g)/(mg/L)^1/n^) and the adsorption intensity factor (n) were calculated by fitting the linear plot of ln (q_e_) by ln (C_e_). The D-R model parameters for the maximum theoretical adsorption capacity (q_m_, mg/g) and a constant (K_DR_, mol^2^/kJ^2^) were determined by the slope and intercept of a linear plot of ln (q_e_) against the Polanyi sorption potential (ε, kJ/mol). The Polanyi sorption potential was computed through equilibrium concentration, temperature and gas constant. In this model, K_DR_ is associated with means free energy of adsorption, R is the universal gas constant (J/mol. K or kJ/mol. K) and T is the absolute temperature in K. In order to assess the adsorption property and to understand the possible adsorption mechanism, an evaluation of parameters for each isotherm model was needed.

For the D-R model, TPh (as GA) and the CODsoluble (as O_2_) concentrations were divided by the molecular weights of 170.12 g/mol and 32 g/mol and the units were converted from mg/L to mol/L. Then the concentration was divided by 1000 for uniformity in units for D-R calculations. Necessary conversion of units was crucial to get model parameters of correct dimensions. The mean free energy of adsorption (E, kJ/mol) was also estimated in terms of the D-R model [48]:


$$\begin{array}{c} E=\frac{\text{1}}{\sqrt{\text{2}K_{DR}}} \end{array}$$ (7)

It is the mean energy to move an adsorbate molecule from the bulk solution to the surface of an adsorbent, which gives information on the strength of interactions and possibly the adsorption mechanism. Physical adsorption behavior is suggested for E < 8 kJ/mol, while ion-exchange mechanisms are typically assumed for values ranging from 8 to 16 kJ/mol. Increased interactions (values of E > 16 kJ/mol) are typically indicative for chemisorption. Evaluation of on 2 isotherm models fitting the results were calculated by determination coefficient *R*^*2*^ according to Equation (7) [49]:


$$\begin{array}{c} R^{\text{2}}=\text{1}-\frac{\sum {\left(q_{e,exp}-q_{e,cal}\right)}^{\text{2}}}{\sum {\left(q_{e,exp}-q_{e,mean}\right)}^{\text{2}}} \end{array}$$ (8)

Where q_e,exp_ and q_e, calc_ are the experimental and model-calculated equilibrium adsorption capacities (mg/g), respectively, and q_e,mean_ is the mean experimental adsorption capacity (mg/g).

### The combined electrocoagulation-adsorption (ECAD) system

At this stage, the adsorption and electrocoagulation processes were set up through a reactor, functioning as one unit, by adding a specific dose of biochar as a supplementary step to the chosen treatment. The optimum process parameters of previously described EC experiment were used in the combined ECAD system. A total of 12 runs were performed with Al and Fe electrode systems. The amount of biochar necessary was calculated theoretically from the batch adsorption mass balance equation (Equation 9). Based on the residual phenol concentration achieved after phenol removal via EC treatment stage, an adsorbent dosage corresponding to regression from 5–30% removal was used. The required dosage of biochar for the combined process was then obtained by rearranging Equation (9) [48]:


$$\begin{array}{c} \frac{v}{m}=\frac{q}{c_{i}-c_{f}} \end{array}$$ (9)

## Assessment of the ECAD treatment operating cost

An important economic parameter which determines the viability and real-life applicability of the wastewater treatment methods is the operating cost (OC). Hence, the operating cost for the combined ECAD treatment was calculated in this work. Although the total cost of treatment involves electrodes, OP biochar, chemicals, electrical energy, labor, sludge dewatering and disposal, and maintenance, only the costs of electrodes, biochar and electrical energy were considered in this study analysis. These ingredients were chosen since they are the main consumable resources related to the ECAD treatment process directly. The OC was calculated by a formula that is widely utilized in the previous studies [50–52]:


$$\begin{array}{c} OC=a\times ENC+b\times ELC+c\times D_{biochar} \end{array}$$ (10)

Where *ENC* is the energy consumption (kWh/m^3^), *ELC* Is the electrode consumption (kg/m^3^), and *D*_*biochar*_ is the dosage of biochar (kg/m^3^). The parameters *a*, *b*, and *c* denote the unit costs of electrical energy, electrodes and OP biochar, respectively in the terms of USD or the local currency of JD. Every element accounted towards the ECAD treatment overall OC.

The *ENC* and *ELC* were computed by the use of Equations (11) and (12), respectively [53]. These equations determine the electricity consumption to process a single m^3^ of wastewater and the theoretical *ELC* following Faraday’s law. For *ENC*:


$$\begin{array}{c} ENC\left(\frac{kWh}{m^{\text{3}}}\right)=\frac{U_{avg}\times I\times t}{\text{1}\text{0}\text{0}\text{0}\times V} \end{array}$$ (11)

where *U*_*avg*_ is the average voltage applied (V), *I* is the current (A), *t* is the duration of the electrolysis (h), and *V* is the volume of treated wastewater (m^3^). The number 1000 was applied to convert units from watts (W) to kilowatts (kW).


$$\begin{array}{c} {ELC}_{theoretical}\left(\frac{kg}{m^{\text{3}}}\right)=\frac{Mw\times I\times t}{n\times F\times v} \end{array}$$ (12)

The theoretical *ELC* was calculated from Faraday’s law where *M*_*w*_ is the molecular weight of the electrode material. The molecular weights of the Al and Fe are 26.98 and 55.85 g/mol, respectively, and were converted to kg/mole for calculations. The t was given in second and the valence of the dissolved metal ions was *n* = 2 for Fe^2+^ and *n* = 3 for Al^3+^ ions, *F* is Faraday’s constant (96,486 C/mol).

In the opinion of Mansouri et al. [54], electrode dissolution, undergoes consumption is the major contribution in degradation of anode, oxidation process causes declining of anode in addition to dissolving metal ions into the wastewater. While the cathode, serves largely as a site for reduction reactions and experiences limited dissolutions. Based on the literature, it is noted that the actual consumption of electrodes in the EC technique is usually higher than the theoretical values calculated according to Faraday's law, but the theoretical values will only be calculated because the actual weight of the electrodes isn’t measured in this study.

Operating resource prices used the analysis were obtained from the sources in specialized October 2025, except for the cost of the OP biochar. The medium-sized industrial firms electricity tariff was 0.068 JD/kWh (around USD 0.096/kWh), the source being the Energy and Minerals Regulatory Commission (EMRC). The costs for electrodes were calculated based on the market prices of aluminium and iron respectively. The global average market price of aluminium was around USD 2,770/ton, according to the data furnished by the London Metal Exchange (LME) [55], while the related price of iron was around USD 570/ton, according to the figures from Trading Economics [56]. Since OP biochar is yet to be priced in the market because of lack of commercial information and data, a cost of USD 0.149/kg quoted by Polonio et al. [57] was considered for the economic study. These unit costs were then added into the operating cost equation to calculate the percentages electrical energy use, electrode consumption and biochar dosages over the total cost of the ECAD process.

## Results and discussion

### Biochar properties

The properties of the biochar obtained from OMSW at 600 °C for 5 h are listed in Table 1. The biochar yields 18.18%, reflecting considerable mass loss during pyrolysis, which is to be expected as volatiles and thermally labile materials are decomposed and evaporated at high temperature. The low moisture content of 4.18% demonstrated the low residual water content of the produced biochar, while the ash content of 10.83% was an indication of the existence of inorganic mineral material in the biochar after pyrolysis.

**Table 1:** Physiochemical properties and structure characteristics of biochar produced at 600°C for 5 hours.

Parameters	Unit	Value
Moisture Content	%	4.18
Electrical Conductivity	mS/cm	0.077
Yield	%	18.18
Ash Content	%	10.83
Zeta Potential	mV	−29.89
Average Pore Width	Å	21.95
Total Pore Volume	cm^3^/g	0.0055
BET Surface Area	m^2^/g	135.23
Langmuir Surface Area	m^2^/g	14.84
“Single point Surface area at P/Po = 0.297”	m^2^/g	9.91
Carbon (C)	%	67.21
Oxygen (O)	%	26.544
Hydrogen (H)	%	3.123
Nitrogen (N)	%	3.123
Atomic ratio H/C	–	0.047
Atomic ratio O/C	–	0.395
Atomic ratio (O + N)/C	–	0.441

As illustrated in Table 1, the multiwalled biochar had a BET surface area of 135.23 m^2^/g, with the average pore width and total pore volume being 21.95 Å and 0.0055 cm^3^/g, respectively. The mean pore width is equivalent to about 2.2 nm and is therefore in the mesoporous range. The formation of a detectable porous structure coupled with a relatively high specific surface area is a desirable property for adsorptive purposes as it enables more accessible surface for contacts between adsorbate and dissolved pollutants. Nevertheless, surface area alone does not define its adsorbent capacity, as this could be influenced by surface charge, pore accessibility, as well as chemical interactions between adsorbent and adsorbate [58].

The disparity between the surface-area values reported is also appreciable. The BET surface area was 135. 23 m^2^/g and the Langmuir and single-point surface areas were 14.84 and 9.91 m^2^/g, respectively (Table 1). The zeta potential of the biochar was −29.89 mV which suggested the biochar surface was negatively charged at the pH of measurement. This negative surface charge can affect how the biochar interacts with dissolved species and may play a role in adsorption via electrostatic interactions, which would be dependent on the charge and chemical configuration of the adsorbate.

The elemental analysis given in the Table 1 revealed that carbon was the major compound in the biochar (67.21%), followed by oxygen (26.544%). Hydrogen and nitrogen were equally detected at 3.123%. The relatively large amount of carbon ensured that the material was carbon-rich after pyrolysis. The atomic ratios were 0.047 H/C, 0.395 O/C and 0.441 (O + N)/C. The low H/C ratio is consistent with the production of a highly carbonized material by pyrolysis and the O/C ratio suggests that oxygen containing compounds were present in the obtained biochar. Overall, the results indicate that pyrolysis of OMSW at 600 °C for 5 h produced a carbon-rich material with a BET surface area of 135.23 m^2^/g, a measurable porous structure, and a negatively charged surface. These characteristics are favorable for its application as an adsorbent.

### FTIR analysis

The FTIR spectra of the raw OMSW and the biochar derived at 600 °C are shown in Fig 1. The raw OMSW showed number of characteristic absorption peaks at 2921 and 2852 cm^-1^ which are predominantly aliphatic C–H stretching and the peak at 1711 cm^-1^ band is a C = O stretching band that maybe derived from carbonyl containing group like carboxylic acid, ester, aldehydes and ketones. The 1638 and 1577 cm^-1^ bands were attributed to aromatic C = C as well as other conjugated structures of lignocellulosic materials. The 1370, 1253, 1031 cm^-1^ band were primarily associated with C–H and C–O moieties. The bands of absorption at 871 and 417 cm^-1^ can be also attributed to the aromatic and the mineral phases, respectively [59].

**Fig 1:**
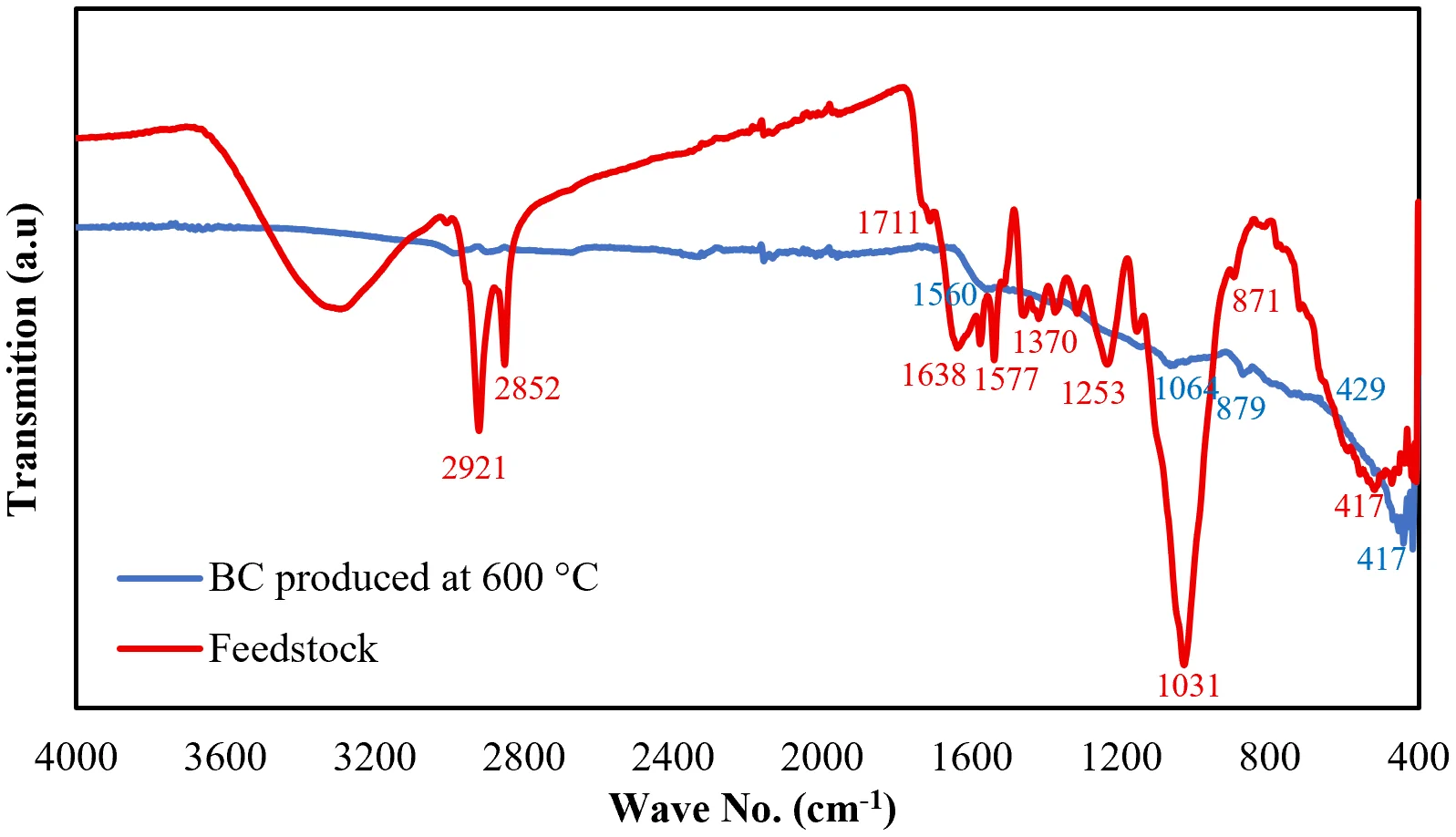
FTIR spectra of raw feedstock (OMSW) and OP biochar produced at 600°C for 5 h.

After thermal treatment at 600 °C the raw OMSW retained most bands disappeared, which suggested that the original organic matter was greatly decomposed and changed during pyrolysis. Sharp features were observed in the biochar spectrum at 1560, 1064, 879, 429, and 417 cm^-1^. The 1560 cm^-1^ band is assigned to aromatic C = C vibrations and 1064 cm^-1^ band is a sign of C–O-containing structures. This agrees well with the chemical analysis of Table 1 where carbon and oxygen were 67.21% and 26.544% respectively with O/C atomic ratio of 0.395. Coupled with disappearance of aliphatic C–H bands the H/C atomic ratio of 0.047 (Table 1) indicates the extensive carbonization and loss of hydrogenated organic structures in pyrolysis. The bands 879, 429 and 417 cm^-1^ can relate to aromatic/inorganic structures and the pair for the lowest wavenumbers may express vibrations of inorganic materials. This is in line with the 10.83% ash in Table 1. In conclusion, FTIR results signify a definite change of raw OMSW to a more carbonized biochar after pyrolysis at 600 °C, but a few oxygenated structures on the biochar surface still survived.

## XRD analysis of biochar

The X-ray diffraction is an effective technique for examining the structural organization and crystallinity of BC derived from the thermal decomposition of OMSW [60]. In this study, X-ray diffraction profiles of the raw OMSW and the biochar obtained at 600 °C for 5 h are shown in Fig 2.

**Fig 2:**
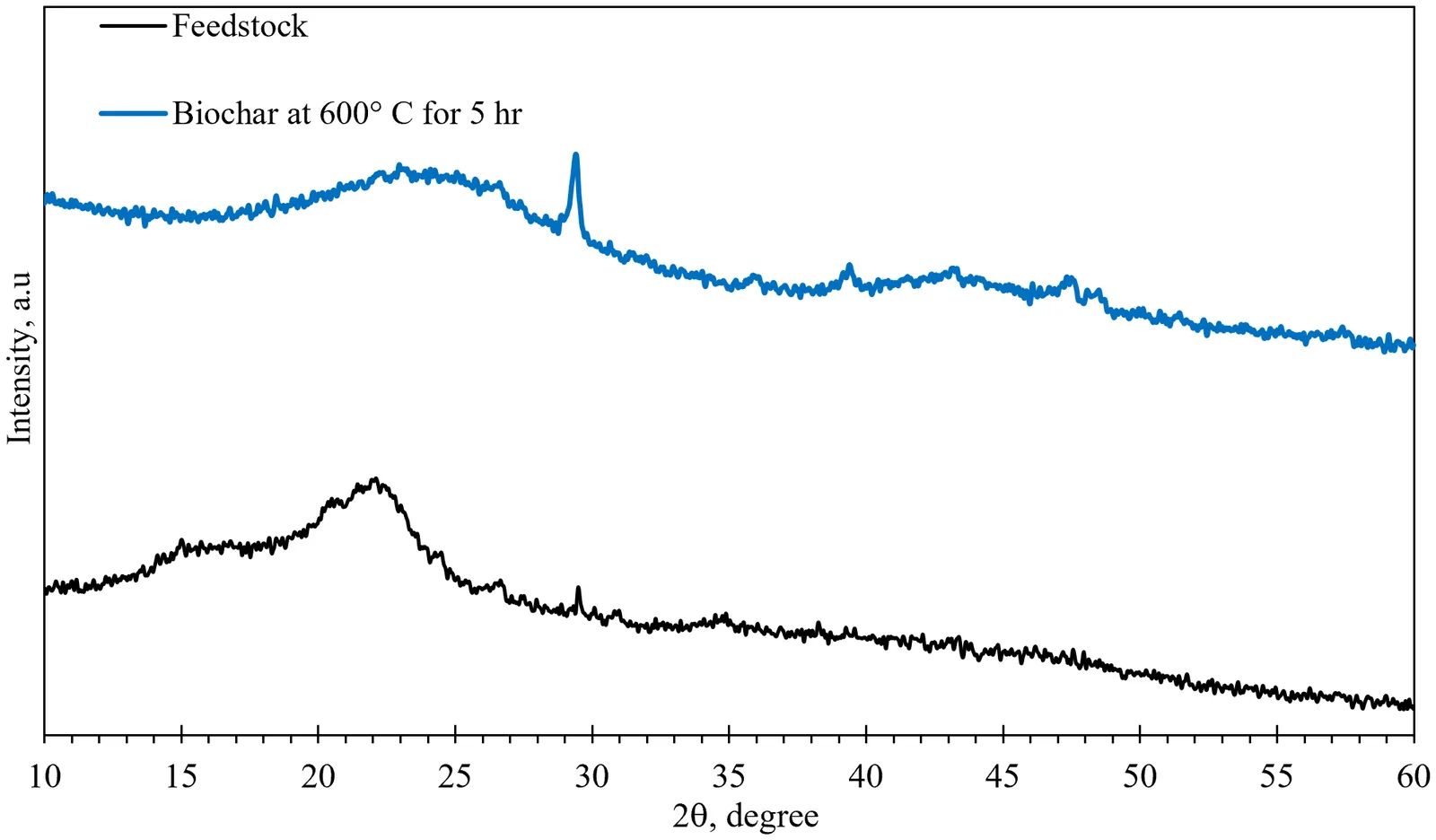
XRD patterns of raw OMSW and OP biochar produced at 600°C for 5 h.

The XRD analysis of the raw OMSW revealed a broad diffraction peak at about 2θ = 15–35°, suggesting an amorphous nature with some crystalline materials [61]. The biochar after pyrolysis at 600 °C had many additional characteristic diffraction peaks near 2θ = 26.7°, 29.4°, 36.0°, 39.4°, 43.0°, 47.5°, 48.4°, 50.0°, and 51.3° besides the broad peak around 2θ = 23°. The appearance of those contact reflections proves the product undergoes a transformation to a more structurally ordered carbonaceous substance during pyrolysis.

This evolution of diffraction features is in line with both the thermal degradation of the original biomass and the synthesis of more condensed carbon materials. The additional reflections can be attributed also to crystalline inorganic compounds formed in the biochar as “ash” % of 10.83% in Table 1. In summary, these XRD results show that the largely amorphous OMSW structure was converted into a more structured and carbonized material containing crystalline inorganic phases after pyrolysis at 600 °C.

### SEM analysis of biochar

The surface topography of the raw OMSW and the biochar at 600 °C for 5 h was analyzed by SEM (Fig 3).

**Fig 3:**
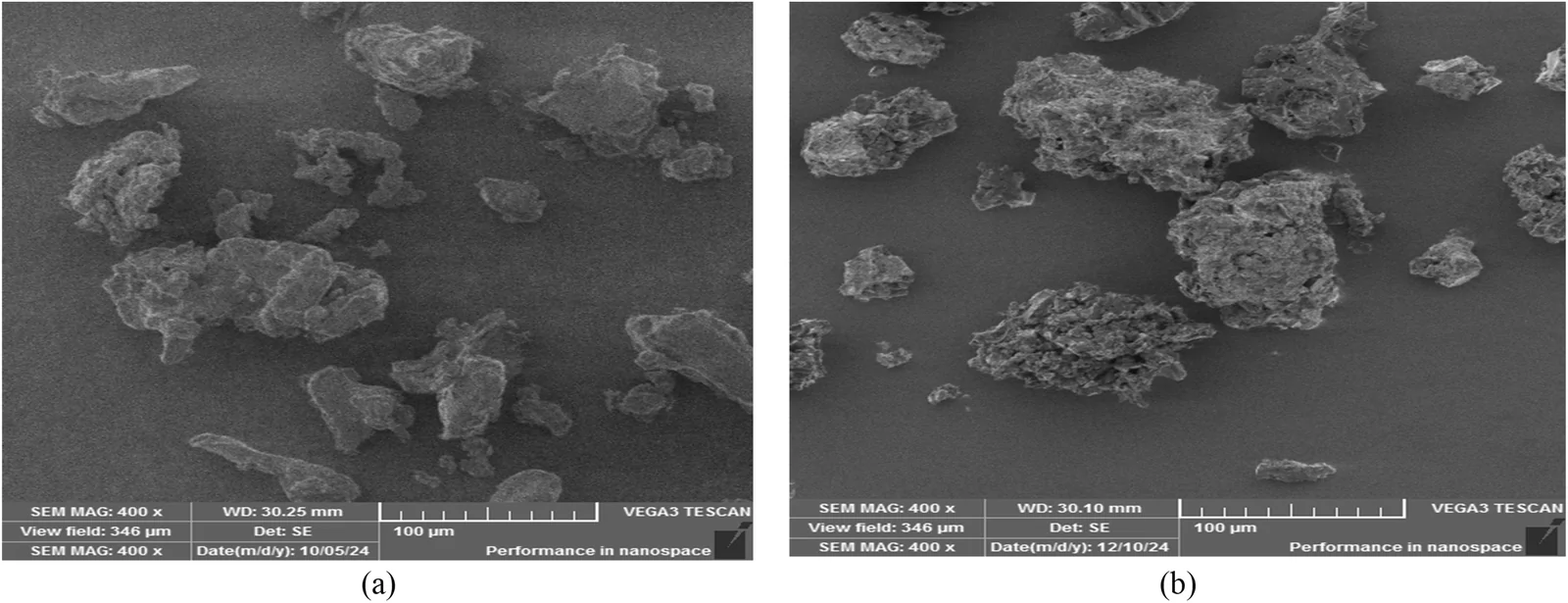
SEM micrographs of (a) raw feedstock and (b) OP biochar produced at 600°C for 5 h.

An irregular and fairly tight surface with few visible pores is shown for the OMSW in the Fig 3(a) raw material. This is the typical morphology of raw biomass, and implies a relatively less-developed porous texture. However, at 600 °C for 5 h (Fig 3(b)), the biochar exhibited a more heterogeneous and enlarged porous surface with visibly larger pores. The occurrence of pores and surface irregularities may be explained by the factor of thermal decomposition and elimination of volatile matters during pyrolysis. This finding is in good agreement with BET surface area of 135.23 m^2^/g in Table 1 and indicative of the formation of accessible surface structure after pyrolysis.

Overall, the results of SEM reveal a distinct morphological difference between the raw and pyrolyzed OMSW, from a relatively compact arrangement at raw OMSW to a well-developed porous structure at 600 °C. The structural properties of the obtained biochar are enhanced by the observed morphological changes together with the surface area and pore size measurements, suggesting that it could be promising as adsorbent material for further application of adsorption sorption. This finding is consistent with the results of Abid et al. [45], who reported that BC produced at 650°C exhibited improved porosity and more visible pores.

### Initial OMW characteristics

The initial properties of OMW in this study are shown in Table 2. Since the raw OMW contains a large number of suspended solids, the samples were filtered through a cloth to remove the coarse solids particles before the experiments of treatment. This filtration was carried out for the convenience of handling the wastewater and not for treatment purpose. Its elevated COD reflects the presence of various soluble organics, including phenolic compounds, sugars, and organic acids, which also contribute to its dark coloration [62,63].

**Table 2:** Characteristics of the raw OMW and pretreated OMW samples.

Parameters	Unit	Raw OMW	Pretreated OMW
Color	–	Light brown	Dark brown
PH	–	3.92	4.4
Turbidity	NTU	1138	349
TSS	mg/L	35,000	2900
COD_total_	mg/L	92,500	67,800
COD_soluble_	mg/L	71,700	67,000
TPh	mg/L	1111.55	944.62
Electrical conductivity	mS/cm	9.2	9.7

As shown in Table 2, the OMW was very acidic (initial pH = 3.92) and had a high electrical conductivity (9.2 mS/cm). With COD_total_ and COD_soluble_ equal to 92,500 and 71,700 mg/L, the wastewater was also highly laden with organics. The TPh value was 1111.55 mg/L, which is an indication of the considerable presence of phenolic compounds. The high turbidity (1138 NTU) and TSS (35,000 mg/L) verified the heavy particulate matter of the wastewater. After cloth filtration, turbidity and TSS reduced to 349 NTU and 2,900 mg/L, respectively, and COD_total_ diminished to 67,800 mg/L. By contrast, COD_soluble_ and TPh were still pretty high with values of 67,000 and 944.62 mg/L, respectively. This means that filtration removed suspended and coarse particles, while a significant amount of dissolved organic and phenolic material in the waste water was retained. The comparatively minor decrease in COD_soluble_ and TPh in contrast to the substantial decline in TSS indicates that the largest proportion of these targeted pollutants existed in the dissolved state.

## Non-linear plots of isotherm models

Non-linear plots of (a) Langmuir, (b) Freundlich and (c) D–R isotherm models obtained in the current study for TPh and OP biochar. The TPh and COD_soluble_ adsorption data on OP biochar were fitted by the nonlinear expressions of the Langmuir, Freundlich and D–R isotherm models, which are shown in Figs 4 and 5.

**Fig 4:**
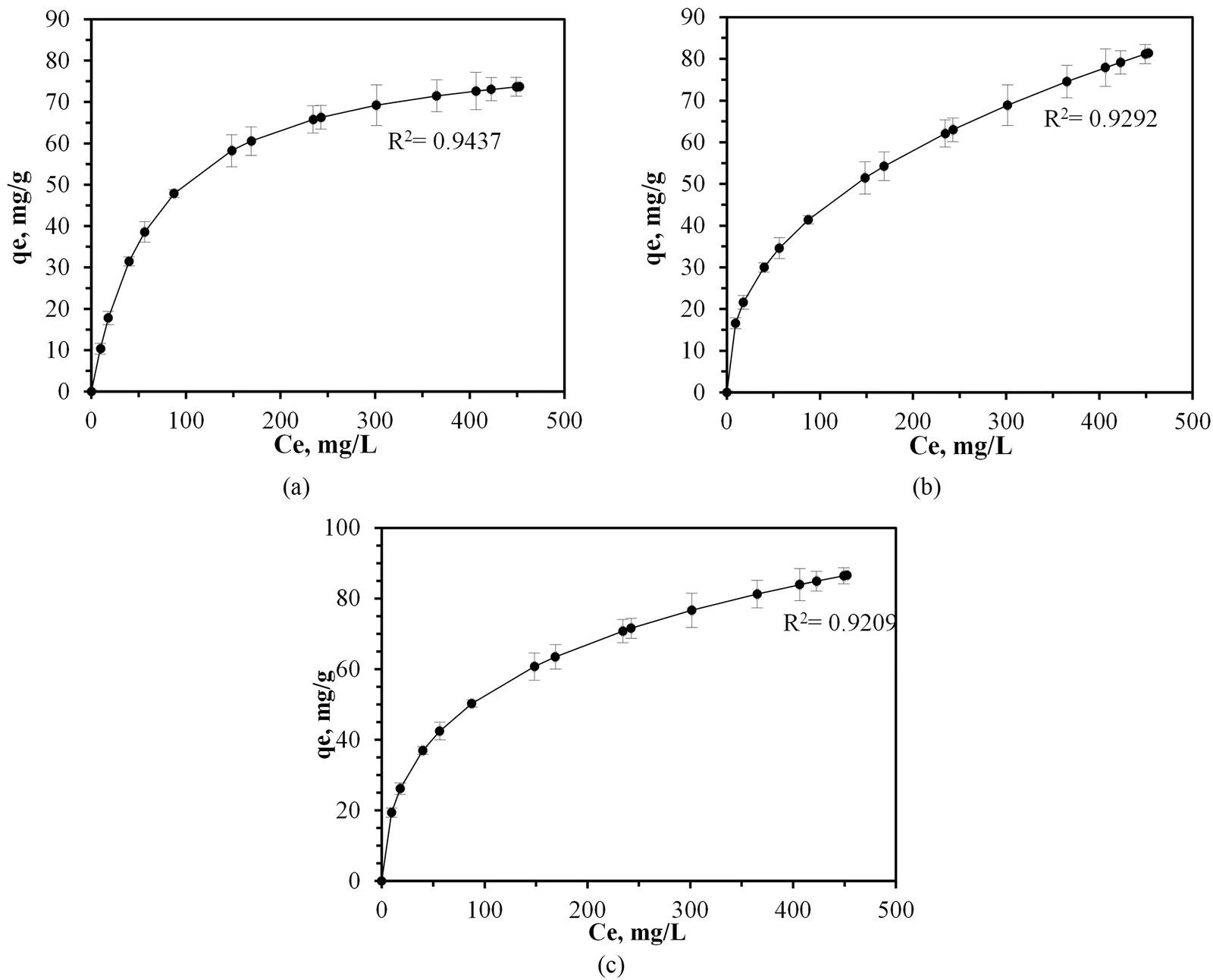
Non-linear fitting of (a) Langmuir, (b) Freundlich, and (c) D-R isotherm models for TPh adsorption onto OP biochar.

**Fig 5:**
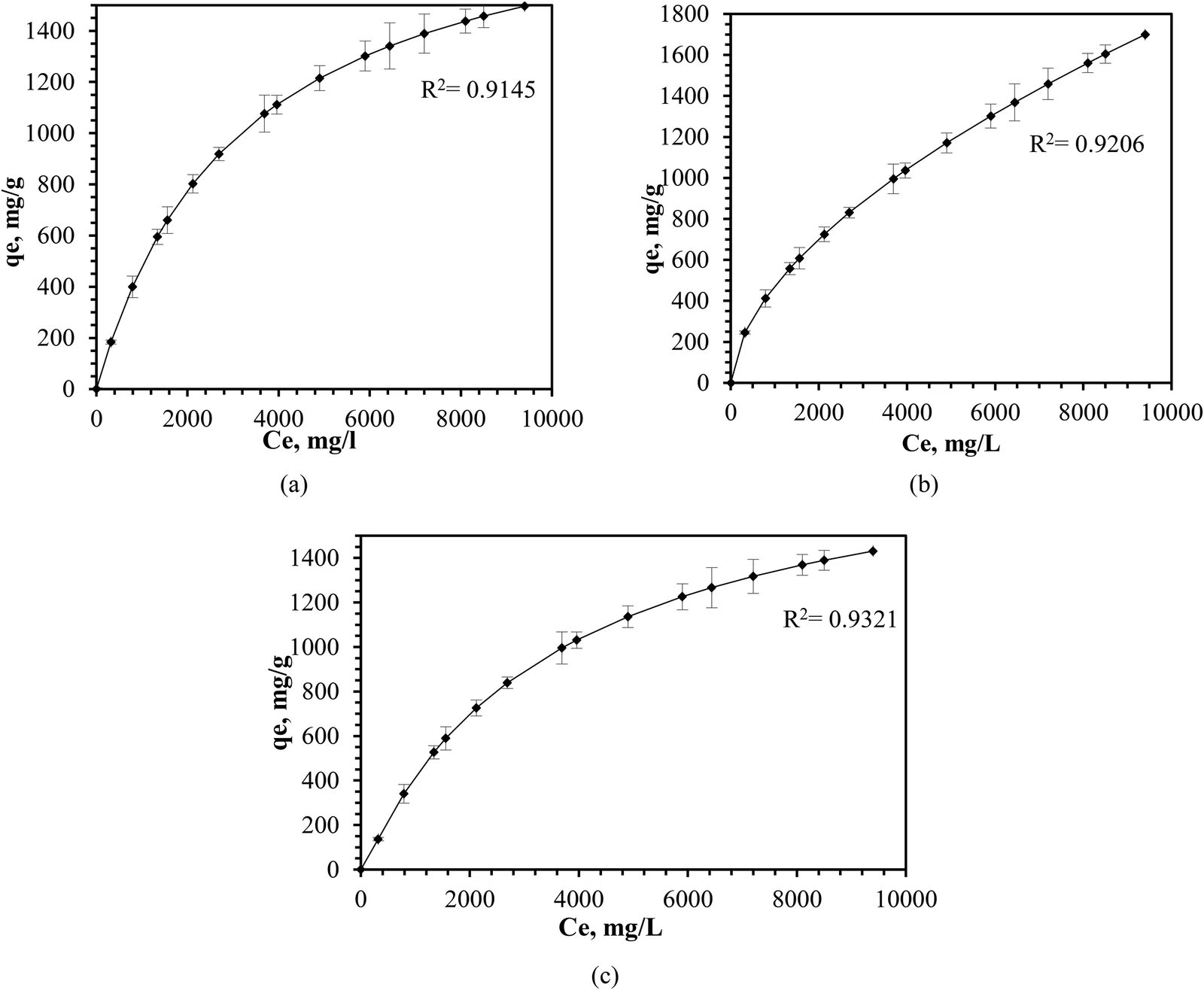
Non-linear fitting of (a) Langmuir, (b) Freundlich, and (c) D-R isotherm models for COD_soluble_ adsorption onto OP biochar.

Fig 4 shows the nonlinear profiles of the isotherms for the TPh adsorption on OP biochar. A rapid increase of the qe as a function of low Ce in the Langmuir model (Fig 4a) is observed, and then the curve is clearly and nonlinearly moving toward a horizontal plateau with increasing Ce values. The adsorption capacity tends to saturate near 74 mg/g for Ce about 300 mg/L, which means the gradual filling of a limited number of adsorption sites registered on the surface of biochar. The Langmuir model fitted the TPh data best, R^2^ = 0.9437.The Freundlich model in (Fig 4b) shows a slower growth rate with increasing qe as the concentration increases in that range, with no obvious saturation plateau in sight for the TPh. The model yields an adsorption capacity of about 81 mg/g at the maximum Ce considered and an R^2^ value of 0.9292. The Freundlich model also yielded a good fit to the experimental results, but not as good as the Langmuir model.

A similar trend was observed for the D–R model (Fig 4c) since it also indicates a gradual increase in the sorption capacity without a clear end part of the curve, the maximum adsorption amount is about 86 mg/g at the highest C e value. The D–R model gave an R^2^ = 0.9209 which was lower than the R^2^ values of the Langmuir and Freundlich models. Generally, the higher R^2^ and obvious saturation trend suggest that Langmuir model is the best model to describing that the TPh adsorption. Fig 5 shows the nonlinear isotherm for the adsorption of COD_soluble_ in OP biochar within a relatively much larger concentration range.

The Langmuir isotherm (Fig 5a) presents a rapid increase of qe at the beginning, and then slowly converges to the higher adsorption capacity with the increase of Ce. At the maximum tested equilibrium concentrations of around 9500 mg/L, the adsorption capacity is about 1480 mg/g. However, a clearly defined saturation plateau is not reached in the investigated concentration range. The Langmuir model gave an R^2^ of 0.9145. The Freundlich model (Fig 5b) has a monotonously increasing adsorption isotherm without a saturation plateau as predicted from the adsorption capacity of around 1700 mg/g at the highest Ce. The model fit had an R^2^ value of 0.9206, indicating that the model described the experimental COD_soluble_ data well, and also slightly better than the Langmuir model.

The D–R curve (Fig 5c) also reveals that the adsorption capacity increases progressively, up to ~1430 mg/g at the maximum equilibrium concentration. Among the three models, the D–R model yielded the maximum coefficient of determination with the R^2^ value of 0.9321 for adsorption of COD_soluble_. This means that the D-R model was the best fit to the experimental data. The loss of a qualitatively defined saturation plateau and increase in adsorption capacity for the entire range of investigated concentrations is in line rather superior fitting of the data by D-R model. The relatively smaller error bars observed in the entire Ce range further imply that the experiments were of low variance and the adsorption data herein were well reproducible. In general, the nonlinear isotherm analysis implies that TPh adsorption is more analogous to a finite-site, saturation-like adsorption behavior as evidenced by the higher fitting of Langmuir model. However, COD_soluble_ adsorption could be well fitted by D–R model, which suggested a wider adsorption nature and no obvious saturated region was observed in the concentration range investigated.

### Fitting of linear isotherm models

Adsorption behavior was quantitatively assessed by using Langmuir, Freundlich, and D–R isotherm models, which were linearized and the respective model parameters were evaluated. For TPh and COD_soluble_ adsorption on OP biochar, the linear isotherm for TPh is given in Fig 6 and for COD_soluble_ is given in Fig 7.

**Fig 6:**
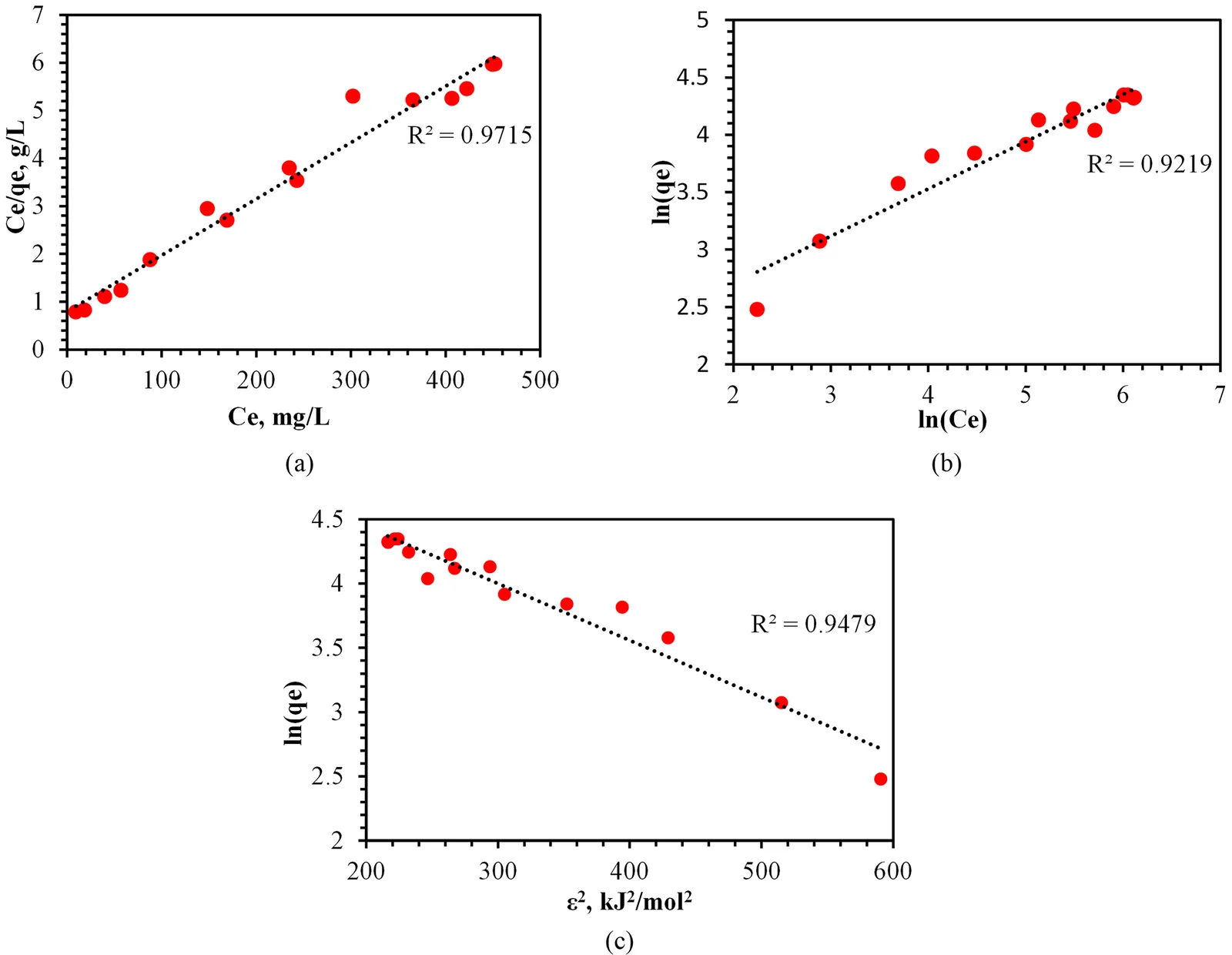
Linearized isotherm plots of (a) Langmuir, (b) Freundlich, and (c) D-R models for TPh adsorption onto OP biochar.

**Fig 7:**
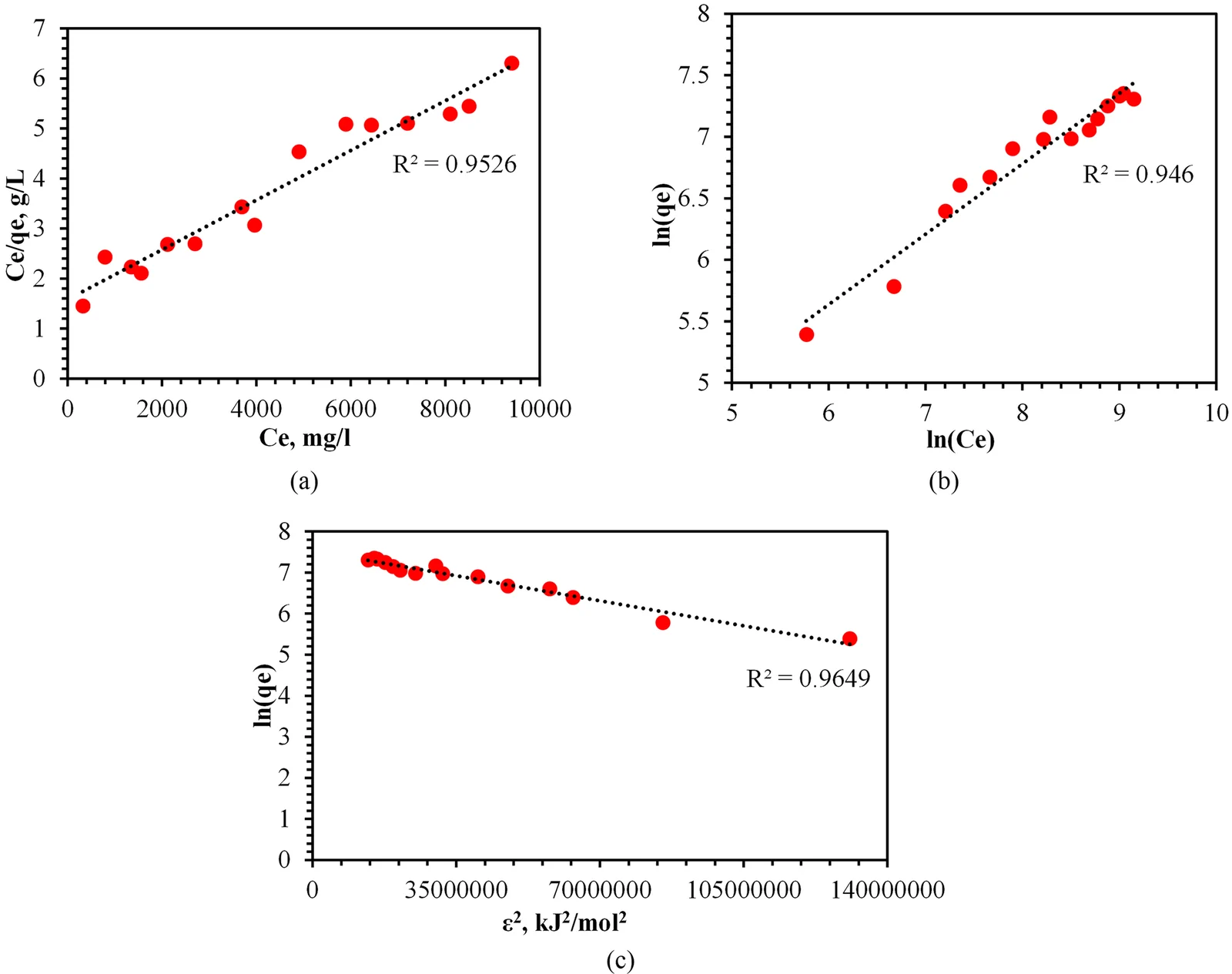
Linearized isotherm plots of (a) Langmuir, (b) Freundlich, and (c) D-R models for COD_soluble_ adsorption onto OP biochar.

The Langmuir model (Fig 6a) was found to best describe the experimental data, with the highest R^2^ of 0.9715 suggesting strong linear relationship. The Freundlich model (Fig 6b) obtained an R^2^ of 0.9219, whereas the D–R model (Fig 6c) had an R^2^ of 0.9479. Consequently, for the quantification of TPh adsorption, the Langmuir model gave the best fit results in the three linearized isotherm models. The superior performance of the Langmuir model implies that TPh adsorption on OP biochar mainly involves the adsorption of a limited number of possibly homogeneous adsorbing sites, and the adsorption process may approach monolayer coverage on the surface. This finding agrees with the nonlinear isotherm model results where the Langmuir model was the best fit to the experimental TPh data.

Fig 7 shows the linear isotherm for COD_soluble_ adsorption on OP biochar.

The Langmuir model (Fig 7a) has the R^2^ value of 0.9526 and the Freundlich model (Fig 7b) has the R^2^ value of 0.9460. The D–R model (Fig 7c) exhibited the maximum degree of goodness of fit with R^2^ value of 0.9649. Hence, the D–R model best described the experimental COD_soluble_ adsorption results. The higher R^2^ value of D–R model implies that adsorption of COD_soluble_ is more accurately described by a model of adsorption energies distribution on biochar surface rather than a constant adsorption energy. Such behavior can be related to the fact that OP biochar surface is non uniform and organic dissolved compounds can bind different kinds of adsorption sites and at the sometime penetrate the existing microporous and carbonaceous structures. Analogous behavior has been found with adsorption processes on heterogeneous biochar surfaces [63–65]. Moreover, the estimated parameters of the three isotherm equations are shown in Table 3.

**Table 3:** Isothermal models parameters.

Models	Langmuir		Freundlich		Dubinin-Radushkevich	
Constants	q_m_, mg/g	K_L_, L/mg	1/n	K_F_, (mg/g)/(mg/L)^1/n^	K_DR_, mol^2^/kJ^2^	E, kJ/mol
TPh	84.74	0.0148	0.41	6.579	4	11.18
COD_soluble_	2000	0.00032	0.57	9.062	2	5

As in Table 3, the q_max_ calculated from the Langmuir model for TPh was 84.74 mg/g for OP biochar. Mohamed Abdoul-Latif et al. [66] studied a comparable OP biochar formation for the elimination of TPh from OMW, though their biochar was formed at a pyrolysis temperature of 600 °C for 5 h. The qm they reported was lower than the value in the present study, which indicates the fairly high adsorption capacity of the OP biochar used in this study. The enhanced adsorption capacity could also be related to the pyrolysis conditions, which determine the specific surface area, pore size distribution and accessibility to phenolic compounds.

Meanwhile, El Hanandeh et al. [67] found a qm of 103.1 mg/g for a chemically activated OP biochar with a FeCl_3_ solution, which was higher. Chemical activation may allow for the creation of more micropores and adjacent active adsorption sites, which can improve the adsorption capability compared to non-activated biochar. Concerning COD_soluble_, an essential D-R model parameter, the E, can be obtained. The estimated E was 5 kJ/mol, which is in the range general for physical adsorption. It also indicates that the OP biochar removal of COD_soluble_ is mainly controlled by relatively weak forces, including physical attraction, intragranular diffusion or pore filling rather than strong chemical binding. The value of 1/n for TPh was 0.41 and 0.57 for COD_soluble_ in the Freundlich model. In both cases (1/n < 1) this indicates good adsorption of the TPh and the COD_soluble_ onto OP biochar [68]. The lower 1/n value for TPh also indicates a favorable adsorption process and predicts a stronger adsorption intensity compared to COD_soluble_ under the tested conditions.

### Electrocoagulation performance of electrode materials

The removal efficiency of the EC process as a single treatment for the elimination of TPh and COD_soluble_ from OMW was investigated with both Al and Fe electrodes. The reported optimal process parameters in literature were used to examine the performance of each electrode and to confirm the derived removal efficiencies. Fig 8 show the removal efficiency of the target contaminant by the Fe- EC and Al-EC processes.

**Fig 8:**
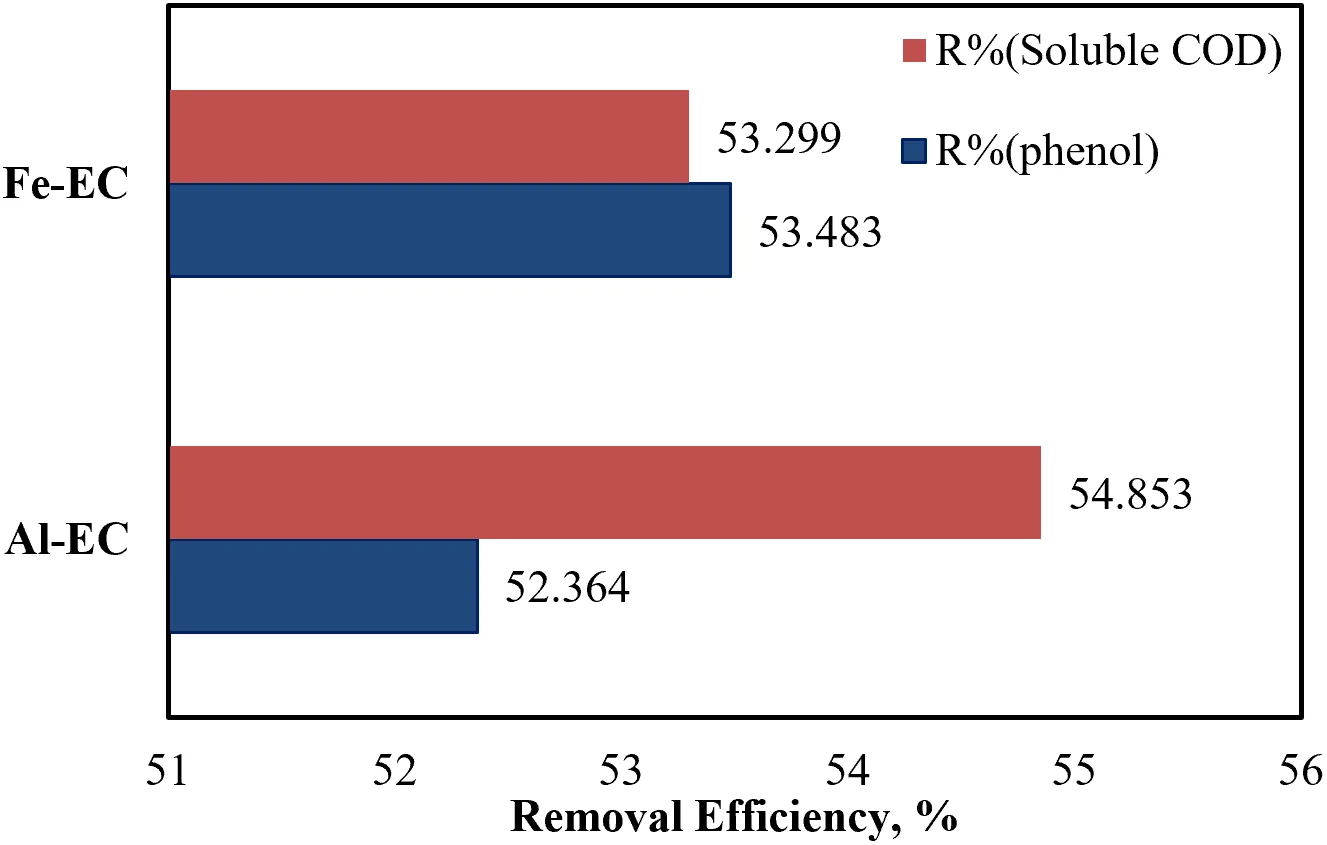
Removal efficiencies of TPh and COD_soluble_ achieved by Al-EC and Fe-EC treatment.

As can be seen in Fig 8, the Al-EC process efficiency for TPh removal was 52.364%, the Fe-EC process had a removal efficiency of 53.483%, which is slightly higher. For COD_soluble_, the removal efficiencies acquired by Al and Fe electrodes were 54.853% and 53.299%, respectively. These results are indicative that both electrode materials could efficiently remove the target contaminates in OMW with their own best operating conditions.The removal efficiencies obtained in this study were comparable with those of Jamrah et al. [5,6] who studied the treatment of the same type of wastewater under similar optimum operating conditions.

In addition, according to the results of TPh and COD_soluble_, the relatively small difference between the removal efficiencies achieved by Al and Fe electrodes implies that both electrodes are almost equal in performance when acting under their best working condition. For TPh, Fe had a slightly better removal efficiency than aluminum while Al had a slightly better removal efficiency for COD_soluble_. Additionally, there was no clear winner in terms of electrodes in both cases which implies that both Al-EC and Fe-EC methods are expected to deliver similar removal performance for TPh and COD_soluble_ in OMW when operated in optimal conditions.

### Theoretical determination of the optimum biochar dosages

A total of 12 theoretical biochar dosages were determined for addition to the EC reactor, based on the primary objective of using biochar as a complementary treatment step to the EC process. Rather than determining the optimum biochar dosage through a large number of independent batch adsorption experiments, the required dosages were theoretically estimated using a mass balance approach combined with the Langmuir isotherm model, which provided the best fit for TPh adsorption. TPh was selected as the basis for determining the biochar dosage rather than COD_soluble_ because TPh has a specific regulatory limit among the minimum contaminant requirements considered for OMW treatment. Therefore, achieving the desired reduction in TPh was used as the primary criterion for estimating the required amount of biochar following the EC treatment.

The final TPh concentrations obtained after the individual EC processes were used as the initial concentrations for the theoretical biochar adsorption calculations. Based on the removal efficiencies obtained previously, the corresponding target TPh concentrations were determined. These target concentrations were then incorporated into the mass balance calculations and the Langmuir adsorption relationship to estimate the biochar mass required to achieve the desired removal level. For the Al-EC process, six theoretical biochar dosages were obtained: 0.108, 0.221, 0.343, 0.474, 0.617, and 0.774 g per 1500 mL of OMW. Similarly, six dosages were calculated for the Fe-EC process: 0.1827, 0.372, 0.569, 0.777, 0.995, and 1.228 g per 1500 Ml.

These theoretically calculated dosages are relatively low compared with the biochar dosages commonly applied in conventional adsorption studies [27,62,69]. The relatively low doses were intentionally selected to maintain the role of biochar as a supplementary component of the ECAD treatment rather than as the primary treatment medium. Using excessive amounts of biochar could introduce undesirable effects, including increased turbidity, interference with electrode operation, and additional color in the treated wastewater. Therefore, the theoretical dosage approach provides a practical means of identifying suitable biochar quantities while minimizing the amount of biochar required for the integrated ECAD process.

## Combined ECAD system

The efficiency of the EC process coupled with OP biochar (ECAD system) was studied by applying EC with dosage of OP biochar at the theoretically calculated dosage. Influence of electrode material (Al and Fe) on the decontamination of TPh and COD_soluble_ was also studied. The removal efficiencies for Al and Fe electrodes are shown in Figs 9 and 10, respectively.

**Fig 9:**
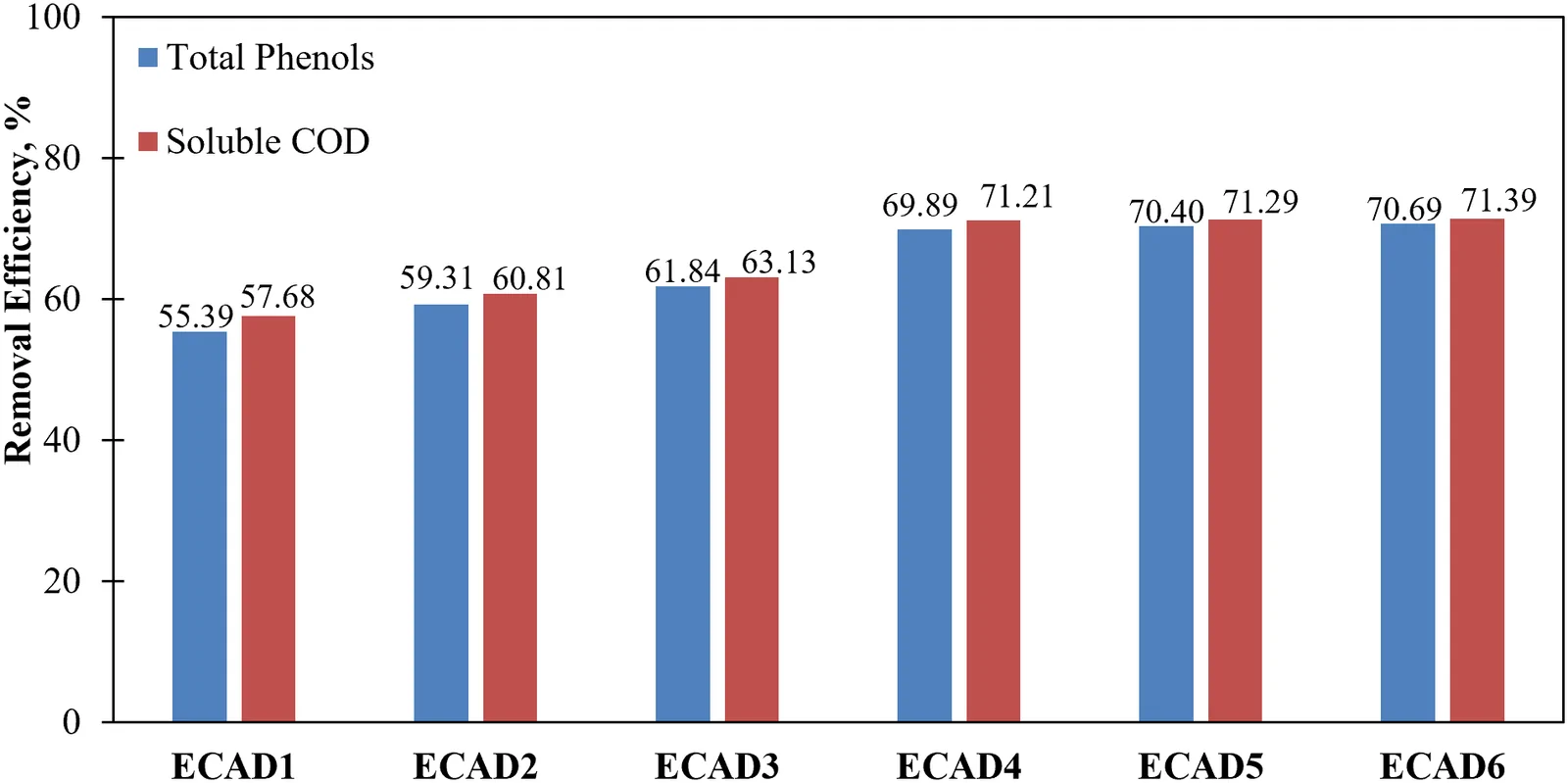
TPh and COD_soluble_ removal efficiencies of the combined Al-ECAD system at different OP biochar dosages.

**Fig 10:**
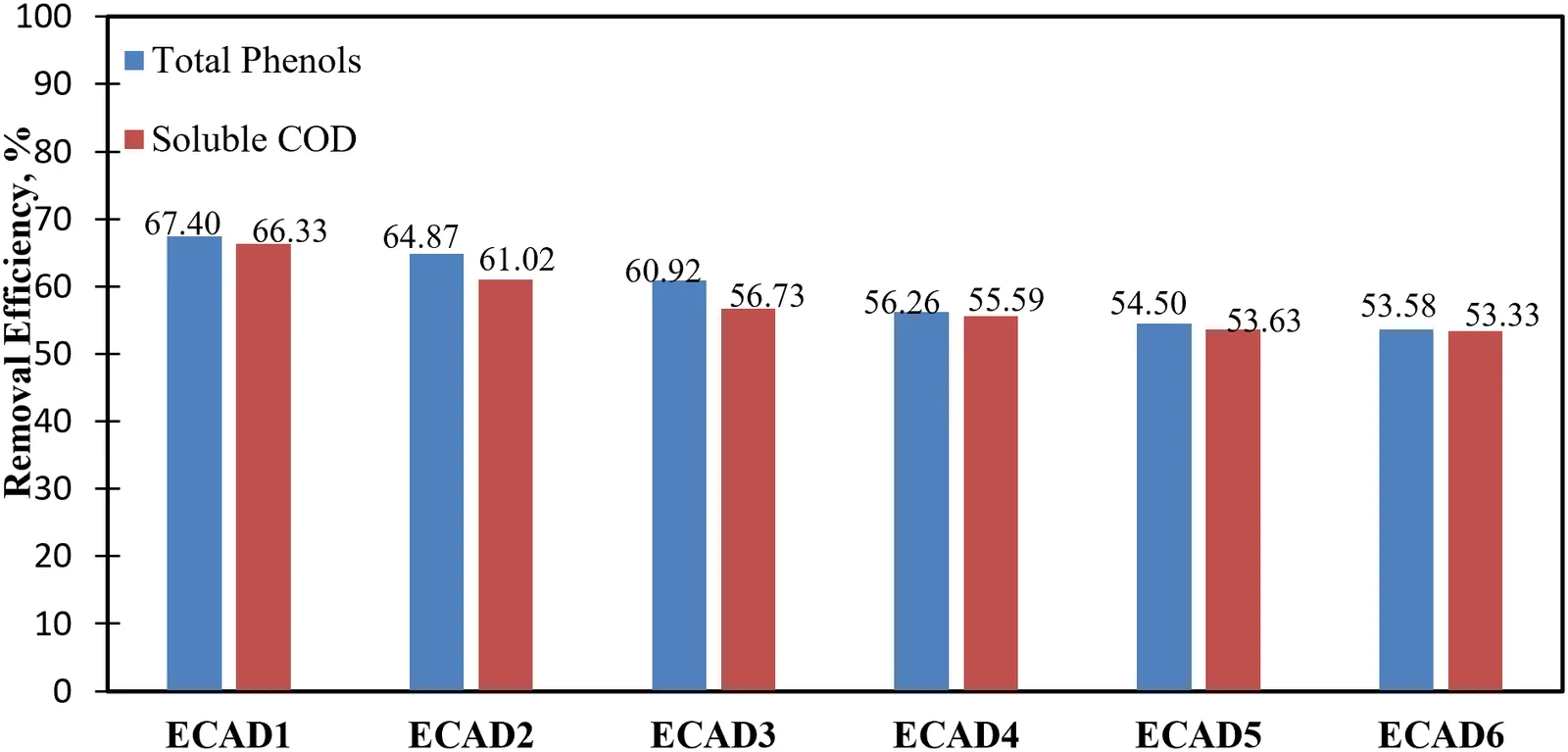
TPh and COD_soluble_ removal efficiencies of the combined Fe-ECAD system at different OP biochar dosages.

The removal efficiencies of the Al-ECAD system are shown in Fig 9. The results indicated a definite improvement in pollutant removal after mixing OP biochar with Al compared to the EC treatment alone. The combination of the two processes mechanisms proved more effective in removing both target pollutants. The maximum treatment efficiencies attained were 70.69% for TPh and 71.39 for COD_soluble_, implying the advantageous effect of biochar in Al based EC. The enhanced performance can be attributed to the complementary action of coagulation and adsorption. EC promotes the destabilization and removal of dissolved and suspended organic compounds through the formation of electrochemically generated coagulant species, while the biochar provides additional adsorption sites and porous structures for the retention of residual organic compounds. Therefore, combining the two mechanisms resulted in higher overall removal efficiencies than those obtained using EC alone.

In contrast, Fig 10 shows a different behavior for the Fe-ECAD system. Although the additional of OP biochar improved the removal efficiency at the lowest investigated biochar dosage, increasing the biochar dosage did not result in a further improvement in contaminant removal. At the lowest biochar dose, the removal efficiencies reached 67.40% for TPh and 66.33% for COD_soluble_. At higher biochar dosages, however, the removal efficiencies decreased, indicating that the additional biochar did not provide a proportional increase in adsorption performance in the presence of Fe electrodes.

This different behavior between the Al- and Fe-based systems may be related to the interaction between the sacrificial electrode species and the biochar surface. The adsorption results obtained from the isotherm analysis indicate that the removal of TPh and COD_soluble_ involves favorable adsorption, with the Freundlich 1/n values below unity, while the D–R energy value obtained for COD_soluble_ indicates a predominantly physical adsorption mechanism. Accordingly, the availability and accessibility of the biochar pore structure and active adsorption sites are important factors governing the performance of the combined system.

For the Al-based system, the generated aluminum species can contribute to coagulation while the biochar simultaneously provides an additional adsorption phase. The standard reduction potential of Al is highly negative (E° = −1.68 volts) [70], and under the aqueous conditions of the present treatment system, the interaction between Al species and the carbonaceous surface may be less detrimental to the accessibility of the biochar adsorption sites. Thus, coagulation and adsorption can contribute to pollutant removal in a complementary manner, allowing the benefit of biochar addition to increase with dosage within the investigated range.

The behavior observed with iron electrodes may be explained by the greater redox activity of Fe species. Fe can occur in both divalent and trivalent forms and undergoes oxidation–reduction reactions under aqueous conditions [71]. These species may interact with the carbonaceous surface and promote the formation or deposition of Fe hydroxide (Fe(OH)_2_) and Fe oxide (Fe(OH)_3_) species within or around the biochar pore structure. Such interactions could partially cover or block accessible adsorption sites and reduce pore accessibility. Previous studies have reported interactions between Fe species and carbonaceous surfaces, including redox exchange between iron ions and carbon materials [72] and the reduction of Fe^3+^ to Fe^2+^ on biochar surfaces [73]. Therefore, progressive interaction or deposition of Fe-containing species may explain why increasing the biochar dosage did not produce the same improvement observed in the Al-ECAD system.

The contrasting responses of the two electrode systems demonstrate that the effectiveness of biochar addition to an EC process is not determined solely by the amount of biochar added. Instead, the interaction between the electrode material, electrochemically generated species, and biochar surface must also be considered. In the present study, the Al-ECAD system showed a clear synergistic improvement with increasing biochar addition, whereas the Fe-ECAD system exhibited its highest performance at the lowest investigated biochar dosage. This electrode-dependent behavior has not been previously reported for an OP-biochar-based ECAD system. Previous studies combining EC with adsorptive materials have generally reported improved pollutant removal compared with EC alone [74,75], but have not specifically evaluated how the sacrificial anode material influences the effectiveness of the combined treatment. The present findings therefore highlight electrode selection as an important consideration when designing and optimizing hybrid ECAD systems for OMW treatment.

### Operation cost of the integrated ECAD system

The total operational cost of the integrated processing, which combines the adsorption and electrocoagulation processes, was estimated for both the aluminum and iron systems. The calculations were based on the actual current and average voltage values applied in each system. The voltages were 14.36 V and 15.11 V for Al and Fe setups, respectively. Taking into account the dose of biochar that achieved the highest removal efficiency for each system. Meanwhile, it is important to emphasize that the cost is approximate and theoretical since the actual electrode loss was not measured, and not all the doses used in the experiments were included. The results showed that the estimated operational cost of each experiment was 4.25 $/m^3^ for the aluminum and 5.30 $/m^3^ for the iron systems. The overall cost of the iron system was higher due to the larger energy consumption. Moreover, these outcomes demonstrate that the combined ECAD remediation can be applied economically to treat the OMW successfully.

## Conclusions

This study demonstrated the feasibility of integrating OP biochar with EC to improve the treatment of OMW, while also demonstrating that the effectiveness of the integrated ECAD process depends strongly on the electrode material. OP biochar produced from OMSW at 600 °C for 5 h exhibited characteristics favorable for adsorption, including a BET surface area of 135.23 m^2^/g, an average pore width of 21.95 Å, a total pore volume of 0.0055 cm^3^/g, and a negative zeta potential of −29.89 mV. The biochar contained 67.21% carbon and showed a low H/C atomic ratio of 0.047, confirming a highly carbonized structure. FTIR analysis indicated the development of aromatic carbon structures with residual oxygen-containing functional groups, while XRD demonstrated the transformation of the largely amorphous raw biomass into a more structured carbonaceous material containing crystalline inorganic phases. SEM further confirmed the development of a more heterogeneous and porous surface after pyrolysis.

The adsorption experiments demonstrated that OP biochar was capable of removing both TPh and COD_soluble_ from OMW. For TPh, the Langmuir model provided the best nonlinear and linear fits, with R^2^ values of 0.9437 and 0.9715, respectively, and a qm of 84.74 mg/g. The Freundlich (1/n) value of 0.41 indicated favorable adsorption. For COD_soluble_, the D–R model provided the best fit, with nonlinear and linear (R^2^) values of 0.9321 and 0.9649, respectively. The corresponding Freundlich (1/n) value of 0.57 also indicated favorable adsorption, while the D–R mean adsorption energy of 5 kJ/mol was consistent with predominantly physical adsorption. These results indicate that TPh adsorption exhibited a stronger tendency toward finite-site saturation, whereas COD_soluble_ adsorption occurred over a more heterogeneous distribution of adsorption energies.

Standalone EC using Al and Fe electrodes produced comparable removal efficiencies under their respective optimum conditions. TPh removal reached 52.364% and 53.483% using Al and Fe electrodes, respectively, while COD_soluble_ removal reached 54.853% and 53.299%, respectively. The similarity between these results confirms that both electrode materials were effective when EC was applied alone. However, the introduction of OP biochar revealed a substantial difference between the two electrode systems. The theoretically calculated biochar dosages, derived from a mass balance and the Langmuir relationship for TPh, ranged from 0.108 to 0.774 g/1500 mL for Al-EC and from 0.1827 to 1.228 g/1500 mL for Fe-EC. The Al-ECAD system showed a clear improvement with biochar addition, achieving maximum removal efficiencies of 70.69% for TPh and 71.39% for COD_soluble_. In contrast, the Fe-ECAD system achieved its highest observed removal at the lowest biochar dosage, with 67.40% TPh and 66.33% COD_soluble_ removal, while increasing the biochar dosage resulted in declining removal efficiencies.

The contrasting behavior of the Al- and Fe-based ECAD systems suggests that the benefit of biochar addition is not universal but is governed by the compatibility between the electrode-generated species and the biochar surface. The improved performance of the Al-ECAD system indicates that EC and adsorption can act as complementary removal mechanisms, whereas the decline observed at higher biochar dosages in the Fe-ECAD system may be associated with interactions between iron species and the carbonaceous surface, including the formation or deposition of iron oxide/hydroxide species that can reduce pore accessibility and block active adsorption sites. This electrode-dependent response represents an important finding of the present study and highlights that electrode selection should be considered as a mechanistic design parameter rather than merely an operational choice in biochar-assisted EC.

From an economic perspective, the estimated operational costs were 4.25 US$/m^3^ for the Al-ECAD system and 5.30 US$/m^3^ for the Fe-ECAD system. The lower cost of the Al configuration, together with its superior integrated treatment performance, identifies Al-ECAD as the more favorable configuration under the investigated conditions. Nevertheless, these costs should be considered preliminary because actual electrode consumption was not directly measured and the calculation did not include all experimental biochar dosages or other potential full-scale costs. Overall, the findings establish OP biochar as a promising complementary adsorbent for EC-based OMW treatment and demonstrate that the success of the ECAD approach depends not only on biochar dosage and adsorption capacity but also on electrode–biochar interactions. The identification of this electrode-dependent effect provides a practical criterion for the design and optimization of hybrid ECAD systems and contributes to the development of low-cost and more sustainable approaches for OMW remediation. Further treatment or polishing stages, together with pilot- or full-scale validation and a comprehensive techno-economic assessment, are recommended before application for discharge or agricultural irrigation reuse.
