# Supplementary material for: Electrode performance in hybrid electrocoagulation and biochar adsorption systems for olive mill wastewater treatment
Source: PLoS One. 2026 Oct 26;21(10):e0360811. doi: 10.1371/journal.pone.0360811 (PMC13704822; doi:10.1371/journal.pone.0360811)
Supplement: Supplementary file 1 [file pone.0360811.s002.docx]

Dear Editor;

Thank you for your efforts. I would like to thank the reviewers for their comments. We considered all of the comments, and we hope that the manuscript (Manuscript PONE-D-26-24570) now fulfills all journal requirements and is suitable for publication. Besides, all the updated parts in the manuscript were highlighted to be easily tracked by the editor and reviewers. Please find an itemized response to the reviewer’s comments:

**Reviewer 1:**

This study investigates the application of combined adsorption and electrocoagulation system for the treatment of olive mill wastewater by comparing the utilization of aluminum and iron electrodes. Many works have been done to achieve the aim of this study. However, the authors need to conduct several adjustment and alignment in order to ensure the manuscript is ready for publication.

Comment 1: Title: - The title needs to be improved to reflect the aim and novelty of this study. Currently, the title looks like a typical experimental works. - The word synergy needs to be clarified or replaced.

Response 1: Thank you for your comment. The title has been revised to "Electrode Performance in Hybrid Electrocoagulation and Biochar Adsorption Systems for Olive Mill Wastewater Treatment." The word "synergy" and its derivatives have been removed and replaced it throughout the manuscript.

Comment 2: Abstract: - Please start the first sentence with a problem statement. - The aim of this study needs to be specified clearly. - Please proposed at least three objectives. - Streamline the major findings only. - State the novelty of this study.

Response 2: Thank you for your comment. The abstract has been rewritten.

Comment 3: Introduction: - The authors need to specify the major problem statements which lead to the foundation of this study. Although the authors did mention olive oil history, it is crucial to justify the needs of such study to be conducted.

Response 3: Thank you for your comment. The Introduction has been revised. The revised text now emphasizes the large volume and highly polluted nature of OMW, its high COD, BOD, and phenolic concentrations, low biodegradability, and the associated environmental risks of inadequate disposal. The specific challenges of OMW management in Jordan, particularly under conditions of water scarcity and an arid climate, were also highlighted.

Comment 4: Introduction: The authors need to cite the four primary steps in processing olive oil. Focus on the production of wastewater.

Response 4: Thank you for your comment. The four primary stages of olive oil processing were described and referenced in Lines (49-54).

Comment 5: Introduction: - Do not start sentence with “Because” line 46.

Response 5: Thank you for your comment. The sentence beginning with "Because" was revised and restructured.

Comment 6: Introduction: - Please synthesize on the disadvantage of the conventional treatment of OMW. Why is it not recommended? How is the process not sustainable?

Response 6: Thank you for your comment. The revised Introduction now provides a more critical synthesis of the limitations of conventional OMW treatment methods.

Comment 7: Introduction: - In terms of electrocoagulation treatment system, the authors need to synthesize more on the existing studies in the literature. The authors are recommended to refer to several studies which are as follows. Sustainable water circularity using electrocoagulation system for Leptobarbus Hoevenii aquaculture wastewater treatment in rural Borneo; Salinity Reduction of Sarawak Brackish Peat Water Sources by Utilizing Electrocoagulation Treatment System with Enhanced Faradaic Efficiency; Corrosion resistance and electrochemical adaptation of aluminum in brackish peat water sources under seawater intrusion in the rural tropical peatlands of Borneo; Unveiling challenges of aluminum electrode fouling and passivation in electrocoagulation treatment system for sustainable water management of coastal Borneo peatlands: A; Kinetic modelling of peat water treatment with continuous electrocoagulation using aluminium electrodes.

Response 7: Thank you for your comment. The Introduction was revised to strengthen the discussion of previous EC studies and to better relate the existing literature to the present work [28-29][38-40].

Comment 8: Introduction: - The authors need to clarify the research gaps in terms of the uniqueness of the proposed study. Despite the idea of proposing EC-adsorption, the authors must also state their novelty. Last sentence of the introduction should mention the novelty of the proposed study.

Response 8: Thank you for your comment. The research gap and novelty of the study were clarified in the revised Introduction.

Comment 9: Introduction: - Effluent Standards and Legislation. This section needs to be integrated into the whole introduction section.

Response 9: Thank you for your comment. The section concerning effluent standards and legislation was integrated into the main Introduction rather than being presented as a separate section.

Comment 10: Methods and Methods – Please check the duplicate word.

Response 10: Thank you for your comment. This has been corrected to "Materials and Methods."

Comment 11: -Sampling and Pretreatment of OMW – The authors are suggested to include the sampling point through map with clear and searchable coordinates.

Response 11: Thank you for your comment. The sampling location and its searchable GPS coordinates have been added to the revised manuscript (Lines 158–159).

Comment 12: Why are the authors using cloth filters? Proper justification and citation are required.

Response 12: Thank you for your comment. A citation has been added justifying the cloth-filter pretreatment step, referencing previously published protocol [23] in lines 165-172, which used an identical procedure.

Comment 13: - Analytical Measurements – The authors are advised to explain “Several steps were in place to ensure the precision data.”

Response 13: Thank you for your comment. This section has been revised (Lines 195-205).

Comment 14: - Measurement of Physicochemical Characteristics. Specify the standard method for each water parameter.

Response 14: Thank you for your comment. This section has been revised and the method used was determined (Lines 183-192).

Comment 15: - Determination of Soluble COD. Briefly describe the procedure described by the manufacturer. - Determination of Total Phenols. Please cite the methods clearly.

Response 15: Thank you for your comment. Thank you for your comment. This section has been revised and the method used was determined (Lines 183-192).

Comment 16: - Preparation of Adsorbent (Biochar). Cite the methods and steps accordingly.

## Response 16: Thank you for your comment. A new section (Olive Mill Solid Waste Sampling and Preparation of Biochar) has been added explaining how to collect and prepare biochar in the laboratory (Lines 206-221).

Comment 17: - The adsorption isotherm experiment. Advisable to illustrate the experimental setup with correct labels.

Response 17: Thank you for your comment. This section has been revised.

Comment 18: The author needs to justify the shaker rotation speed.

Response 18: Thank you for your comment. The shaker speed of 150 rpm was justified as providing uniform biochar suspension and effective dispersion while minimizing mass-transfer limitations and avoiding potential particle damage, based on the literature [49–51]. This justification was added in Lines 271–273.

Comment 19: - Plotting Adsorption Isotherms Curve Models. Please include R2 to study the regression of the proposed models.

Response 19: Thank you for your comment. R^2^ values have been added.

Comment 20: - The EC experiment. Should be placed before adsorption of isotherm models.

Response 20: Thank you for your comment. The "Electrocoagulation Experiment" section has been moved before the "Adsorption Isotherm Experiment" section (Page 10 ).

Comment 21: - The Combined ECAD System. Please write in fill name.

Response 21: Thank you for your comment. The ECAD has been rewritten in its full name (line 325).

Comment 22: Cite Equation 1.

Response 22: Thank you for your comment. The equation number was updated to Equation 2, and all equations have been properly cited in the manuscript.

Comment 23: Results and Discussion: - The authors need to critically synthesize their findings in accordance with the literature review. Such actions will outstand their findings will eventually strengthen their novelty.

Response 23: Thank you for your comment. The Results and Discussion section was refined.

Comment 24: - The findings need to be aligned with Method section.

Response 24: Thank you for your comment. This Section has been revised.

Comment 25: Conclusions: - First sentence needs to highlight what has been done in executing the experiment. - The authors need to streamline the major findings based on the proposed objectives. - Last sentence must statement the knowledge contribution to the body of literature.

Response 25: Thank you for your comment. The conclusions have been rewritten.

Comment 26: References: - The authors need to ensure the year of citation is from 2020 onwards.

Response 26: Thank you for your comment. The references were reviewed and updated, and recent references published from 2020 onwards were used wherever possible. Older references were retained only where they were necessary.

**Reviewer 2:**

The manuscript addresses an interesting topic by combining olive pomace biochar with electrocoagulation for olive mill wastewater treatment. However, substantial revision is required before the manuscript can be considered for publication.

Comment 1: The abstract should be rewritten to be more attractive, concise, and scientifically engaging. At present, it reports results but does not clearly highlight the novelty, research gap, practical significance, or key contribution of the study.

Response 1: Thank you for your comment. The Abstract has been revised.

Comment 2: Introduction claims that the proposed combined treatment is cost-effective, environmentally friendly, and highly effective. However, these claims are not sufficiently supported by comparative evidence, cost analysis, environmental assessment, or direct comparison with other treatment methods. The authors should provide clearer justification and supporting references or data.

Response 2: Thank you for your comment. Introduction has been revised. A dedicated cost analysis section has been included in the manuscript.

Comment 3: The experimental methodology is incomplete. The number of replicates is insufficient; measurements should be performed in more than triplicate where appropriate. Standard deviation, error bars, and statistical analysis should be included to support the reliability of the results. The manuscript should also provide calibration curves, raw data, method validation details, and clearer quality control information.

Response 3: Thank you for your comment. The experimental methodology has been revised and clarified in the manuscript to provide more detailed information.

Comment 4: The authors should clarify ethical or permission issues related to the collection and use of olive mill wastewater samples. If field sampling was conducted, relevant permission or institutional approval should be stated.

Response 4: Thank you for your comment. The OMW samples were collected from the selected olive mills under an official authorization letter issued by the university and addressed to the respective mills. The samples were collected solely for research purposes with the permission of the relevant facilities. This clarification has been added to the revised manuscript (Lines 164-166).

Comment 5: The characterization of the biochar is insufficient. Additional analyses such as BET surface area, pore size distribution, SEM, FTIR, elemental composition, or other relevant characterization should be included to better explain the adsorption mechanism and support the discussion.

Response 5: Thank you for your comment. The biochar characterization has been substantially expanded in the revised manuscript. The requested analyses, including BET surface area and pore characteristics, SEM, FTIR, and elemental composition, have been added and discussed to better support the interpretation of the adsorption behavior. The characterization procedures were also clearly described in the “Olive Mill Solid Waste Sampling and Preparation of Biochar” and “Biochar Characterization” sections (Lines 206–235). The corresponding characterization results and their implications for the adsorption mechanism are presented in the “Biochar Properties” (Lines 383–417), “FTIR Analysis” (Lines 418–442), “XRD Analysis of Biochar” (Lines 443–462), and “SEM Analysis of Biochar” (Lines 463–482) sections.

Comment 6: The figures require major improvement. Figure 4 appears to repeat information already presented in Figures 2 and 3. Most graphs lack SD or error bars. The x- and y-axes are not well organized, and the figure quality, resolution, and formatting should be improved.

Response 6: Thank you for your comment. All Figures have been reorganized and improved. Figure 4 has been removed to clear redundancy. All Figures have been reorganized and improved.

Comment 7: Figures 7, 8, and 9 are not presented in the most appropriate way. Different parameters such as total phenols and soluble COD should not be compared in a single unclear format. The authors should separate the results into clearer graphs, for example one graph for TPh and another for soluble COD, and directly compare Al and Fe electrodes under the same treatment conditions.

Response 7: Thank you for your comment. Figures 8, 9, and 10 were intentionally designed in a combined format to allow a direct, side-by-side comparison between the Al and Fe electrodes for both TPh and COD_soluble_ under identical experimental conditions.

Comment 8: The Discussion section is too limited and does not sufficiently explain the mechanisms, trends, differences between Al and Fe electrodes, biochar interaction, or comparison with previous studies. The authors should substantially expand the discussion and link the findings more clearly to the experimental results.

Response 8: Thank you for your comment. The Results and Discussion section has been expanded significantly.
