# Supplementary material for: Electrode performance in hybrid electrocoagulation and biochar adsorption systems for olive mill wastewater treatment
Source: PLoS One. 2026 Oct 26;21(10):e0360811. doi: 10.1371/journal.pone.0360811 (PMC13704822; doi:10.1371/journal.pone.0360811)
Supplement: Peer review history

# Peer review: Decision Letter 0

**DOI:** 10.1371/journal.pone.0360811.r001

**Authors:** Muammar Qadafi

30 Jun 2026

Dear Dr. Al-Zghoul,

Thank you for submitting your manuscript to PLOS ONE. After careful consideration, we feel that it has merit but does not fully meet PLOS ONE’s publication criteria as it currently stands. Therefore, we invite you to submit a revised version of the manuscript that addresses the points raised during the review process.

Please submit your revised manuscript by Aug 29 2026 11:59PM. If you will need more time than this to complete your revisions, please reply to this message or contact the journal office at plosone@plos.org. When you're ready to submit your revision, log on to https://www.editorialmanager.com/pone/ and select the 'Submissions Needing Revision' folder to locate your manuscript file.

- A letter that responds to each point raised by the academic editor and reviewer(s). You should upload this letter as a separate file labeled 'Response to Reviewers'.
- A marked-up copy of your manuscript that highlights changes made to the original version. You should upload this as a separate file labeled 'Revised Manuscript with Track Changes'.
- An unmarked version of your revised paper without tracked changes. You should upload this as a separate file labeled 'Manuscript'.

If you would like to make changes to your financial disclosure, please include your updated statement in your cover letter. Guidelines for resubmitting your figure files are available below the reviewer comments at the end of this letter.

If applicable, we recommend that you deposit your laboratory protocols in protocols.io to enhance the reproducibility of your results. Protocols.io assigns your protocol its own identifier (DOI) so that it can be cited independently in the future. For instructions see: https://journals.plos.org/plosone/s/submission-guidelines#loc-laboratory-protocols. Additionally, PLOS ONE offers an option for publishing peer-reviewed Lab Protocol articles, which describe protocols hosted on protocols.io. Read more information on sharing protocols at https://plos.org/protocols?utm_medium=editorial-email&utm_source=authorletters&utm_campaign=protocols.

As the corresponding author, your ORCID iD is verified in the submission system and will appear in the published article. PLOS supports the use of ORCID, and we encourage all coauthors to register for an ORCID iD and use it as well. Please encourage your coauthors to verify their ORCID iD within the submission system before final acceptance, as unverified ORCID iDs will not appear in the published article. *Only*  the individual author can complete the verification step; PLOS staff *cannot*  verify ORCID iDs on behalf of authors.

We look forward to receiving your revised manuscript.

Kind regards,

Muammar Qadafi

Academic Editor

PLOS One

Journal Requirements:

When submitting your revision, we need you to address these additional requirements.

1. Please ensure that your manuscript meets PLOS ONE's style requirements, including those for file naming. The PLOS ONE style templates can be found at

https://journals.plos.org/plosone/s/file?id=wjVg/PLOSOne_formatting_sample_main_body.pdf and https://journals.plos.org/plosone/s/file?id=ba62/PLOSOne_formatting_sample_title_authors_affiliations.pdf

2. In your Methods section, please provide additional information regarding the permits you obtained for the work. Please ensure you have included the full name of the authority that approved the field site access and, if no permits were required, a brief statement explaining why.

3. Please provide a complete Data Availability Statement in the submission form, ensuring you include all necessary access information or a reason for why you are unable to make your data freely accessible. If your research concerns only data provided within your submission, please write "All data are in the manuscript and/or supporting information files" as your Data Availability Statement.

4. We note that Figure 1 in your submission contain copyrighted images. All PLOS content is published under the Creative Commons Attribution License (CC BY 4.0), which means that the manuscript, images, and Supporting Information files will be freely available online, and any third party is permitted to access, download, copy, distribute, and use these materials in any way, even commercially, with proper attribution. For more information, see our copyright guidelines: http://journals.plos.org/plosone/s/licenses-and-copyright.

We require you to either (1) present written permission from the copyright holder to publish these figures specifically under the CC BY 4.0 license, or (2) remove the figures from your submission:

a. You may seek permission from the original copyright holder of Figure 1 to publish the content specifically under the CC BY 4.0 license.

We recommend that you contact the original copyright holder with the Content Permission Form (http://journals.plos.org/plosone/s/file?id=7c09/content-permission-form.pdf) and the following text:

“I request permission for the open-access journal PLOS ONE to publish XXX under the Creative Commons Attribution License (CCAL) CC BY 4.0 (http://creativecommons.org/licenses/by/4.0/). Please be aware that this license allows unrestricted use and distribution, even commercially, by third parties. Please reply and provide explicit written permission to publish XXX under a CC BY license and complete the attached form.”

Please upload the completed Content Permission Form or other proof of granted permissions as an "Other" file with your submission.

In the figure caption of the copyrighted figure, please include the following text: “Reprinted from [ref] under a CC BY license, with permission from [name of publisher], original copyright [original copyright year].”

b. If you are unable to obtain permission from the original copyright holder to publish these figures under the CC BY 4.0 license or if the copyright holder’s requirements are incompatible with the CC BY 4.0 license, please either i) remove the figure or ii) supply a replacement figure that complies with the CC BY 4.0 license. Please check copyright information on all replacement figures and update the figure caption with source information. If applicable, please specify in the figure caption text when a figure is similar but not identical to the original image and is therefore for illustrative purposes only.

5. If the reviewer comments include a recommendation to cite specific previously published works, please review and evaluate these publications to determine whether they are relevant and should be cited. There is no requirement to cite these works unless the editor has indicated otherwise.

[Note: HTML markup is below. Please do not edit.]

Reviewers' comments:

Reviewer's Responses to Questions

**Comments to the Author**

1. Is the manuscript technically sound, and do the data support the conclusions?

Reviewer #1: Yes

Reviewer #2: Partly

**********

2. Has the statistical analysis been performed appropriately and rigorously?

Reviewer #1: Yes

Reviewer #2: No

**********

3. Have the authors made all data underlying the findings in their manuscript fully available?

The PLOS Data policy

Reviewer #1: Yes

Reviewer #2: Yes

**********

4. Is the manuscript presented in an intelligible fashion and written in standard English?

Reviewer #1: Yes

Reviewer #2: No

**********

Reviewer #1: Overall Comment.

This study investigates the application of combined adsorption and electrocoagulation system for the treatment of olive mill wastewater by comparing the utilization of aluminum and iron electrodes. Many works have been done to achieve the aim of this study. However, the authors need to conduct several adjustment and alignment in order to ensure the manuscript is ready for publication.

Title

- The title needs to be improved to reflect the aim and novelty of this study. Currently, the title looks like a typical experimental works.

- The word synergy needs to be clarified or replaced.

Abstract

- Please start the first sentence with a problem statement

- The aim of this study needs to be specified clearly.

- Please proposed at least three objectives

- Streamline the major findings only

- State the novelty of this study.

Introduction

- The authors need to specify the major problem statements which lead to the foundation of this study. Although the authors did mention olive oil history, it is crucial to justify the needs of such study to be conducted.

- The authors need to cite the four primary steps in processing olive oil. Focus on the production of wastewater.

- Do not start sentence with “Because” line 46.

- Please synthesize on the disadvantage of the conventional treatment of OMW. Why is it not recommended? How is the process not sustainable?

- In terms of electrocoagulation treatment system, the authors need to synthesize more on the existing studies in the literature. The authors are recommended to refer to several studies which are as follows. Sustainable water circularity using electrocoagulation system for Leptobarbus Hoevenii aquaculture wastewater treatment in rural Borneo; Salinity Reduction of Sarawak Brackish Peat Water Sources by Utilizing Electrocoagulation Treatment System with Enhanced Faradaic Efficiency; Corrosion resistance and electrochemical adaptation of aluminium in brackish peat water sources under seawater intrusion in the rural tropical peatlands of Borneo; Unveiling challenges of aluminium electrode fouling and passivation in electrocoagulation treatment system for sustainable water management of coastal Borneo peatlands: A; Kinetic modelling of peat water treatment with continuous electrocoagulation using aluminium electrodes.

- The authors need to clarify the research gaps in terms of the uniqueness of the proposed study. Despite the idea of proposing EC-adsorption, the authors must also state their novelty. Last sentence of the introduction should mention the novelty of the proposed study.

- Effluent Standards and Legislation. This section needs to be integrated into the whole introduction section.

Methods and Methods – Please check the duplicate word

- Sampling and Pretreatment of OMW – The authors are suggested to include the sampling point through map with clear and searchable coordinates. Why are the authors using cloth filters? Proper justification and citation are required.

- Analytical Measurements – The authors are advised to explain “Several steps were in place to ensure the precision data.”

- Measurement of Physicochemical Characteristics. Specify the standard method for each water parameter.

- Determination of Soluble COD. Briefly describe the procedure described by the manufacturer.

- Determination of Total Phenols. Please cite the methods clearly.

- Preparation of Adsorbent (Biochar). Cite the methods and steps accordingly.

- The adsorption isotherm experiment. Advisable to illustrate the experimental setup with correct labels. The author needs to justify the shaker rotation speed.

- Plotting Adsorption Isotherms Curve Models. Please include R2 to study the regression of the proposed models.

- The EC experiment. Should be placed before adsorption of isotherm models.

- The Combined ECAD System. Please write in fill name. Cite Equation 1.

Results and Discussion

- The authors need to critically synthesize their findings in accordance with the literature review. Such actions will outstand their findings will eventually strengthen their novelty.

- The findings need to be aligned with Method section.

Conclusions

- First sentence needs to highlight what has been done in executing the experiment.

- The authors need to streamline the major findings based on the proposed objectives.

- Last sentence must statement the knowledge contribution to the body of literature.

References

- The authors need to ensure the year of citation is from 2020 onwards.

Reviewer #2: Recommendation to the Authors

The manuscript addresses an interesting topic by combining olive pomace biochar with electrocoagulation for olive mill wastewater treatment. However, substantial revision is required before the manuscript can be considered for publication.

First, the abstract should be rewritten to be more attractive, concise, and scientifically engaging. At present, it reports results but does not clearly highlight the novelty, research gap, practical significance, or key contribution of the study.

Second, the Introduction claims that the proposed combined treatment is cost-effective, environmentally friendly, and highly effective. However, these claims are not sufficiently supported by comparative evidence, cost analysis, environmental assessment, or direct comparison with other treatment methods. The authors should provide clearer justification and supporting references or data.

Third, the experimental methodology is incomplete. The number of replicates is insufficient; measurements should be performed in more than triplicate where appropriate. Standard deviation, error bars, and statistical analysis should be included to support the reliability of the results. The manuscript should also provide calibration curves, raw data, method validation details, and clearer quality control information.

Fourth, the authors should clarify ethical or permission issues related to the collection and use of olive mill wastewater samples. If field sampling was conducted, relevant permission or institutional approval should be stated.

Fifth, the characterization of the biochar is insufficient. Additional analyses such as BET surface area, pore size distribution, SEM, FTIR, elemental composition, or other relevant characterization should be included to better explain the adsorption mechanism and support the discussion.

Sixth, the figures require major improvement. Figure 4 appears to repeat information already presented in Figures 2 and 3. Most graphs lack SD or error bars. The x- and y-axes are not well organized, and the figure quality, resolution, and formatting should be improved.

Seventh, Figures 7, 8, and 9 are not presented in the most appropriate way. Different parameters such as total phenols and soluble COD should not be compared in a single unclear format. The authors should separate the results into clearer graphs, for example one graph for TPh and another for soluble COD, and directly compare Al and Fe electrodes under the same treatment conditions.

Finally, the Discussion section is too limited and does not sufficiently explain the mechanisms, trends, differences between Al and Fe electrodes, biochar interaction, or comparison with previous studies. The authors should substantially expand the discussion and link the findings more clearly to the experimental results.

Overall, I recommend major revision. The study has potential, but the manuscript requires substantial improvement in experimental design, statistical analysis, data presentation, biochar characterization, and scientific discussion before it can be accepted.

**********

what does this mean?). If published, this will include your full peer review and any attached files.

If you choose “no”, your identity will remain anonymous but your review may still be made public.

**Do you want your identity to be public for this peer review?**  For information about this choice, including consent withdrawal, please see our Privacy Policy

Reviewer #1: No

Reviewer #2: No

**********

[NOTE: If reviewer comments were submitted as an attachment file, they will be attached to this email and accessible via the submission site. Please log into your account, locate the manuscript record, and check for the action link "View Attachments". If this link does not appear, there are no attachment files.]

To ensure your figures meet our technical requirements, please review our figure guidelines: https://journals.plos.org/plosone/s/figures

You may also use PLOS’s free figure tool, NAAS, to help you prepare publication quality figures: https://journals.plos.org/plosone/s/figures#loc-tools-for-figure-preparation.

NAAS will assess whether your figures meet our technical requirements by comparing each figure against our figure specifications.

# Author comment: Author response to Decision Letter 1

**DOI:** 10.1371/journal.pone.0360811.r002

20 Sep 2026

Dear Editor;

Thank you for your efforts. I would like to thank the reviewers for their comments. We considered all of the comments, and we hope that the manuscript (Manuscript PONE-D-26-24570) now fulfills all journal requirements and is suitable for publication. Besides, all the updated parts in the manuscript were highlighted to be easily tracked by the editor and reviewers. Please find an itemized response to the reviewer’s comments:

Reviewer 1:

This study investigates the application of combined adsorption and electrocoagulation system for the treatment of olive mill wastewater by comparing the utilization of aluminum and iron electrodes. Many works have been done to achieve the aim of this study. However, the authors need to conduct several adjustment and alignment in order to ensure the manuscript is ready for publication.

Comment 1: Title: - The title needs to be improved to reflect the aim and novelty of this study. Currently, the title looks like a typical experimental works. - The word synergy needs to be clarified or replaced.

Response 1: Thank you for your comment. The title has been revised to "Electrode Performance in Hybrid Electrocoagulation and Biochar Adsorption Systems for Olive Mill Wastewater Treatment." The word "synergy" and its derivatives have been removed and replaced it throughout the manuscript.

Comment 2: Abstract: - Please start the first sentence with a problem statement. - The aim of this study needs to be specified clearly. - Please proposed at least three objectives. - Streamline the major findings only. - State the novelty of this study.

Response 2: Thank you for your comment. The abstract has been rewritten.

Comment 3: Introduction: - The authors need to specify the major problem statements which lead to the foundation of this study. Although the authors did mention olive oil history, it is crucial to justify the needs of such study to be conducted.

Response 3: Thank you for your comment. The Introduction has been revised. The revised text now emphasizes the large volume and highly polluted nature of OMW, its high COD, BOD, and phenolic concentrations, low biodegradability, and the associated environmental risks of inadequate disposal. The specific challenges of OMW management in Jordan, particularly under conditions of water scarcity and an arid climate, were also highlighted.

Comment 4: Introduction: The authors need to cite the four primary steps in processing olive oil. Focus on the production of wastewater.

Response 4: Thank you for your comment. The four primary stages of olive oil processing were described and referenced in Lines (49-54).

Comment 5: Introduction: - Do not start sentence with “Because” line 46.

Response 5: Thank you for your comment. The sentence beginning with "Because" was revised and restructured.

Comment 6: Introduction: - Please synthesize on the disadvantage of the conventional treatment of OMW. Why is it not recommended? How is the process not sustainable?

Response 6: Thank you for your comment. The revised Introduction now provides a more critical synthesis of the limitations of conventional OMW treatment methods.

Comment 7: Introduction: - In terms of electrocoagulation treatment system, the authors need to synthesize more on the existing studies in the literature. The authors are recommended to refer to several studies which are as follows. Sustainable water circularity using electrocoagulation system for Leptobarbus Hoevenii aquaculture wastewater treatment in rural Borneo; Salinity Reduction of Sarawak Brackish Peat Water Sources by Utilizing Electrocoagulation Treatment System with Enhanced Faradaic Efficiency; Corrosion resistance and electrochemical adaptation of aluminum in brackish peat water sources under seawater intrusion in the rural tropical peatlands of Borneo; Unveiling challenges of aluminum electrode fouling and passivation in electrocoagulation treatment system for sustainable water management of coastal Borneo peatlands: A; Kinetic modelling of peat water treatment with continuous electrocoagulation using aluminium electrodes.

Response 7: Thank you for your comment. The Introduction was revised to strengthen the discussion of previous EC studies and to better relate the existing literature to the present work [28-29][38-40].

Comment 8: Introduction: - The authors need to clarify the research gaps in terms of the uniqueness of the proposed study. Despite the idea of proposing EC-adsorption, the authors must also state their novelty. Last sentence of the introduction should mention the novelty of the proposed study.

Response 8: Thank you for your comment. The research gap and novelty of the study were clarified in the revised Introduction.

Comment 9: Introduction: - Effluent Standards and Legislation. This section needs to be integrated into the whole introduction section.

Response 9: Thank you for your comment. The section concerning effluent standards and legislation was integrated into the main Introduction rather than being presented as a separate section.

Comment 10: Methods and Methods – Please check the duplicate word.

Response 10: Thank you for your comment. This has been corrected to "Materials and Methods."

Comment 11: -Sampling and Pretreatment of OMW – The authors are suggested to include the sampling point through map with clear and searchable coordinates.

Response 11: Thank you for your comment. The sampling location and its searchable GPS coordinates have been added to the revised manuscript (Lines 158–159).

Comment 12: Why are the authors using cloth filters? Proper justification and citation are required.

Response 12: Thank you for your comment. A citation has been added justifying the cloth-filter pretreatment step, referencing previously published protocol [23] in lines 165-172, which used an identical procedure.

Comment 13: - Analytical Measurements – The authors are advised to explain “Several steps were in place to ensure the precision data.”

Response 13: Thank you for your comment. This section has been revised (Lines 195-205).

Comment 14: - Measurement of Physicochemical Characteristics. Specify the standard method for each water parameter.

Response 14: Thank you for your comment. This section has been revised and the method used was determined (Lines 183-192).

Comment 15: - Determination of Soluble COD. Briefly describe the procedure described by the manufacturer. - Determination of Total Phenols. Please cite the methods clearly.

Response 15: Thank you for your comment. Thank you for your comment. This section has been revised and the method used was determined (Lines 183-192).

Comment 16: - Preparation of Adsorbent (Biochar). Cite the methods and steps accordingly.

Response 16: Thank you for your comment. A new section (Olive Mill Solid Waste Sampling and Preparation of Biochar) has been added explaining how to collect and prepare biochar in the laboratory (Lines 206-221).

Comment 17: - The adsorption isotherm experiment. Advisable to illustrate the experimental setup with correct labels.

Response 17: Thank you for your comment. This section has been revised.

Comment 18: The author needs to justify the shaker rotation speed.

Response 18: Thank you for your comment. The shaker speed of 150 rpm was justified as providing uniform biochar suspension and effective dispersion while minimizing mass-transfer limitations and avoiding potential particle damage, based on the literature [49–51]. This justification was added in Lines 271–273.

Comment 19: - Plotting Adsorption Isotherms Curve Models. Please include R2 to study the regression of the proposed models.

Response 19: Thank you for your comment. R2 values have been added.

Comment 20: - The EC experiment. Should be placed before adsorption of isotherm models.

Response 20: Thank you for your comment. The "Electrocoagulation Experiment" section has been moved before the "Adsorption Isotherm Experiment" section (Page 10 ).

Comment 21: - The Combined ECAD System. Please write in fill name.

Response 21: Thank you for your comment. The ECAD has been rewritten in its full name (line 325).

Comment 22: Cite Equation 1.

Response 22: Thank you for your comment. The equation number was updated to Equation 2, and all equations have been properly cited in the manuscript.

Comment 23: Results and Discussion: - The authors need to critically synthesize their findings in accordance with the literature review. Such actions will outstand their findings will eventually strengthen their novelty.

Response 23: Thank you for your comment. The Results and Discussion section was refined.

Comment 24: - The findings need to be aligned with Method section.

Response 24: Thank you for your comment. This Section has been revised.

Comment 25: Conclusions: - First sentence needs to highlight what has been done in executing the experiment. - The authors need to streamline the major findings based on the proposed objectives. - Last sentence must statement the knowledge contribution to the body of literature.

Response 25: Thank you for your comment. The Conclusions have been rewritten accordingly.

Comment 26: References: - The authors need to ensure the year of citation is from 2020 onwards.

Response 26: Thank you for your comment. The references were reviewed and updated, and recent references published from 2020 onwards were used wherever possible. Older references were retained only where they were necessary.

Reviewer 2:

The manuscript addresses an interesting topic by combining olive pomace biochar with electrocoagulation for olive mill wastewater treatment. However, substantial revision is required before the manuscript can be considered for publication.

Comment 1: The abstract should be rewritten to be more attractive, concise, and scientifically engaging. At present, it reports results but does not clearly highlight the novelty, research gap, practical significance, or key contribution of the study.

Response 1: Thank you for your comment. The Abstract has been revised.

Comment 2: Introduction claims that the proposed combined treatment is cost-effective, environmentally friendly, and highly effective. However, these claims are not sufficiently supported by comparative evidence, cost analysis, environmental assessment, or direct comparison with other treatment methods. The authors should provide clearer justification and supporting references or data.

Response 2: Thank you for your comment. Introduction has been revised. A dedicated cost analysis section has been included in the manuscript.

Comment 3: The experimental methodology is incomplete. The number of replicates is insufficient; measurements should be performed in more than triplicate where appropriate. Standard deviation, error bars, and statistical analysis should be included to support the reliability of the results. The manuscript should also provide calibration curves, raw data, method validation details, and clearer quality control information.

Response 3: Thank you for your comment. The experimental methodology has been revised and clarified in the manuscript to provide more detailed information.

Comment 4: The authors should clarify ethical or permission issues related to the collection and use of olive mill wastewater samples. If field sampling was conducted, relevant permission or institutional approval should be stated.

Response 4: Thank you for your comment. The OMW samples were collected from the selected olive mills under an official authorization letter issued by the university and addressed to the respective mills. The samples were collected solely for research purposes with the permission of the relevant facilities. This clarification has been added to the revised manuscript (Lines 164-166).

Comment 5: The characterization of the biochar is insufficient. Additional analyses such as BET surface area, pore size distribution, SEM, FTIR, elemental composition, or other relevant characterization should be included to better explain the adsorption mechanism and support the discussion.

Response 5: Thank you for your comment. The biochar characterization has been substantially expanded in the revised manuscript. The requested analyses, including BET surface area and pore characteristics, SEM, FTIR, and elemental composition, have been added and discussed to better support the interpretation of the adsorption behavior. The characterization procedures were also clearly described in the “Olive Mill Solid Waste Sampling and Preparation of Biochar” and “Biochar Characterization” sections (Lines 206–235). The corresponding characterization results and their implications for the adsorption mechanism are presented in the “Biochar Properties” (Lines 387–422), “FTIR Analysis” (Lines 423–447), “XRD Analysis of Biochar” (Lines 448–468), and “SEM Analysis of Biochar” (Lines 469–487) sections.

Comment 6: The figures require major improvement. Figure 4 appears to repeat information already presented in Figures 2 and 3. Most graphs lack SD or error bars. The x- and y-axes are not well organized, and the figure quality, resolution, and formatting should be improved.

Response 6: Thank you for your comment. All Figures have been reorganized and improved. Figure 4 has been removed to clear redundancy. All Figures have been reorganized and improved.

Comment 7: Figures 7, 8, and 9 are not presented in the most appropriate way. Different parameters such as total phenols and soluble COD should not be compared in a single unclear format. The authors should separate the results into clearer graphs, for example one graph for TPh and another for soluble COD, and directly compare Al and Fe electrodes under the same treatment conditions.

Response 7: Thank you for your comment. Figures 9, 10, and 11 were intentionally designed in a combined format to allow a direct, side-by-side comparison between the Al and Fe electrodes for both TPh and CODsoluble under identical experimental conditions.

Comment 8: The Discussion section is too limited and does not sufficiently explain the mechanisms, trends, differences between Al and Fe electrodes, biochar interaction, or comparison with previous studies. The authors should substantially expand the discussion and link the findings more clearly to the experimental results.

Response 8: Thank you for your comment. The Results and Discussion section has been expanded significantly.

Attachment Submitted filename: Response to reviewer v3.docx

# Peer review: Decision Letter 1

**DOI:** 10.1371/journal.pone.0360811.r003

**Authors:** Muammar Qadafi

1 Oct 2026

Electrode Performance in Hybrid Electrocoagulation and Biochar Adsorption Systems for Olive Mill Wastewater Treatment

PONE-D-26-24570R1

Dear Dr. Al-Zghoul,

We’re pleased to inform you that your manuscript has been judged scientifically suitable for publication and will be formally accepted for publication once it meets all outstanding technical requirements.

Within one week, you’ll receive an e-mail detailing the required amendments. When these have been addressed, you’ll receive a formal acceptance letter and your manuscript will be scheduled for publication.

An invoice will be generated when your article is formally accepted. Please note, if your institution has a publishing partnership with PLOS and your article meets the relevant criteria, all or part of your publication costs will be covered. Please make sure your user information is up-to-date by logging into Editorial Manager at Editorial Manager® and clicking the ‘Update My Information' link at the top of the page. For questions related to billing, please contact billing support.

If your institution or institutions have a press office, please notify them about your upcoming paper to help maximize its impact. If they’ll be preparing press materials, please inform our press team as soon as possible -- no later than 48 hours after receiving the formal acceptance. Your manuscript will remain under strict press embargo until 2 pm Eastern Time on the date of publication. For more information, please contact onepress@plos.org.

Kind regards,

Muammar Qadafi

Academic Editor

PLOS One

Additional Editor Comments (optional):

Reviewers' comments:

Reviewer's Responses to Questions

**Comments to the Author**

Reviewer #1: All comments have been addressed

Reviewer #2: All comments have been addressed

**********

2. Is the manuscript technically sound, and do the data support the conclusions?

Reviewer #1: Yes

Reviewer #2: Yes

**********

3. Has the statistical analysis been performed appropriately and rigorously?

Reviewer #1: Yes

Reviewer #2: Yes

**********

4. Have the authors made all data underlying the findings in their manuscript fully available?

The PLOS Data policy

Reviewer #1: Yes

Reviewer #2: Yes

**********

5. Is the manuscript presented in an intelligible fashion and written in standard English?

Reviewer #1: Yes

Reviewer #2: Yes

**********

Reviewer #1: Acceptable in current form. The authors revised the manuscript accordingly. The manuscript is deemed suitable for punlication.

Reviewer #2: The revised manuscript has been substantially improved, and the authors have adequately addressed the major concerns raised during the previous review. In particular, the methodology and experimental procedures are now described more clearly, and additional characterization of the olive pomace biochar has strengthened the scientific basis of the study.

The revised Results and Discussion section provides a clearer interpretation of the adsorption behavior and the performance of the Al- and Fe-based electrocoagulation–adsorption systems. The main findings are generally consistent with the data presented, and the conclusions appropriately reflect the observed differences between the two electrode systems. The authors have also appropriately acknowledged the limitations of the operational cost estimation and the need for further pilot- or full-scale validation.

The clarification regarding permission for wastewater sample collection is appreciated. I also note that the authors have declared no competing interests and no specific funding for this work.

Overall, the revised manuscript is technically sound and has been considerably strengthened in response to the reviewers' comments. I have no further major concerns. Minor proofreading for grammar, wording, and formatting is recommended before publication.

**********

what does this mean?). If published, this will include your full peer review and any attached files.

If you choose “no”, your identity will remain anonymous but your review may still be made public.

**Do you want your identity to be public for this peer review?**  For information about this choice, including consent withdrawal, please see our Privacy Policy

Reviewer #1: No

Reviewer #2: No

**********

# Editor report: Acceptance letter

**DOI:** 10.1371/journal.pone.0360811.r004

**Authors:** Muammar Qadafi

PONE-D-26-24570R1

PLOS One

Dear Dr. Al-Zghoul,

I'm pleased to inform you that your manuscript has been deemed suitable for publication in PLOS One. Congratulations! Your manuscript is now being handed over to our production team.

At this stage, our production department will prepare your paper for publication. This includes ensuring the following:

* All references, tables, and figures are properly cited

* All relevant supporting information is included in the manuscript submission,

* There are no issues that prevent the paper from being properly typeset

You will receive further instructions from the production team, including instructions on how to review your proof when it is ready. Please keep in mind that we are working through a large volume of accepted articles, so please give us a few days to review your paper and let you know the next and final steps.

Lastly, if your institution or institutions have a press office, please let them know about your upcoming paper now to help maximize its impact. If they'll be preparing press materials, please inform our press team within the next 48 hours. Your manuscript will remain under strict press embargo until 2 pm Eastern Time on the date of publication. For more information, please contact onepress@plos.org.

You will receive an invoice from PLOS for your publication fee after your manuscript has reached the completed accept phase. If you receive an email requesting payment before acceptance or for any other service, this may be a phishing scheme. Learn how to identify phishing emails and protect your accounts at https://explore.plos.org/phishing.

If we can help with anything else, please email us at customercare@plos.org.

Thank you for submitting your work to PLOS One and supporting open access.

Kind regards,

PLOS One Editorial Office Staff

on behalf of

Dr. Muammar Qadafi

Academic Editor

PLOS One
